# miR-135b-3p Promotes Cardiomyocyte Ferroptosis by Targeting GPX4 and Aggravates Myocardial Ischemia/Reperfusion Injury

**DOI:** 10.3389/fcvm.2021.663832

**Published:** 2021-08-13

**Authors:** Weixin Sun, Ruijie Shi, Jun Guo, Haiyan Wang, Le Shen, Haibo Shi, Peng Yu, Xiaohu Chen

**Affiliations:** ^1^Department of Cardiology, Yancheng TCM Hospital Affiliated to Nanjing University of Chinese Medicine, Yancheng, China; ^2^Department of Cardiology, Jiangsu Province Hospital of Chinese Medicine, Affiliated Hospital of Nanjing University of Chinese Medicine, Nanjing, China; ^3^First Clinical Medical College, Nanjing University of Chinese Medicine, Nanjing, China; ^4^Department of Cardiology, Liyang City Hospital of TCM, Changzhou, China

**Keywords:** miR-135b-3p, ferroptosis, GPX4, cardiomyocyte ferroptosis, myocardial ischemia/reperfusion injury

## Abstract

Ferroptosis is a form of cell death induced by excess iron and accumulation of reactive oxygen species in cells. Recently, ferroptosis has been reported to be associated with cancer and ischemia/reperfusion (I/R) injury in multiple organs. However, the regulatory effects and underlying mechanisms of myocardial I/R injury are not well-understood. The role of miR-135b-3p as an oncogene that accelerates tumor development has been confirmed; however, its role in myocardial I/R is not fully understood. In this study, we established an *in vivo* myocardial I/R rat model and an *in vitro* hypoxia/reoxygenation (H/R)-induced H9C2 cardiomyocyte injury model and observed that ferroptosis occurred in tissues and cells during I/R myocardial injury. We used database analysis to find miR-135b-3p and validated its inhibitory effect on the ferroptosis-related gene glutathione peroxidase 4 (*Gpx4*), using a luciferase reporter assay. Furthermore, miR-135b-3p was found to promote the myocardial I/R injury by downregulating GPX4 expression. The results of this study elucidate a novel function of miR-135b-3p in exacerbating cardiomyocyte ferroptosis, providing a new therapeutic target for improving I/R injury.

## Introduction

Cardiovascular disease is one of the leading causes of death in humans (1). Myocardial ischemia/reperfusion (I/R) may be a therapeutic approach for protecting against acute myocardial ischemic infarction (MI) (2). However, as a result of direct blood flow restoration to ischemic tissue, myocardial I/R also leads to cell death and additional cell dysfunction. For example, the primary pathological manifestation of coronary artery disease is myocardial I/R injury. Myocardial I/R injury does not recover and is involved in inflammation, calcium overload, oxidative stress, cytokine release, and neutrophil infiltration (3). Therefore, elucidation of the molecular mechanisms of myocardial I/R injury has great significance and clinical value and may provide a potential new target for clinical treatment.

The irreversible damage to the heart and brain following I/R has received much attention in recent years. Many scholars have focused their attention on ferroptosis and have confirmed through their studies that ferroptosis plays an important role in I/R injury (4–6). Ferroptosis, first proposed by Dr. Brent R. Stockwell of Columbia University in 2012, is an iron-dependent, novel form of programmed cell death that is distinct from apoptosis, necrosis, and autophagy (7). Ferroptosis occurs primarily due to the failure of the membrane lipid repair enzyme GPX4, resulting in the accumulation of lipid peroxides and reactive oxygen species (ROS) (5). Cancer cells carrying oncogenic Ras appear to be more sensitive to ferritin induction; therefore, this form of cell death has also been explored for cancer therapy (7, 8). Ferroptosis inhibitors are effective in treating other diseases, such as I/R-induced organ damage in experimental models (9, 10). For example, liproxstatin-1 inhibits ferroptosis and promotes cell survival by reducing voltage-dependent anion channel 1 (VDAC1) levels and restoring GPX4 levels to protect the mouse myocardium against I/R injury (11). The mechanistic target of rapamycin (mTOR) has protective effects against excess iron accumulation and ferroptosis in cardiomyocytes (12). Although ferroptosis is strongly implicated in human myocardial I/R injury, the precise molecular mechanisms and biological functions of ferroptosis remain poorly understood. Therefore, in this study, we conducted an in-depth study of the specific mechanisms underlying the occurrence of ferroptosis in I/R.

miRNAs are a class of endogenous non-coding small RNAs that are widely present in the body, with a length of approximately 21–22 nucleotides (13). Studies have shown that miRNAs participate in various life processes, and their abnormal expression is involved in the occurrence and development of multiple diseases, including myocardial I/R injury (14). Previous studies have shown that the presence of miRNAs can regulate cell survival in response to an I/R injury (15). The role of miRNAs in regulating ferroptosis has been reported in several types of cancer (16) but not in myocardial I/R injury. Since GPX4 plays a crucial role in the onset of ferroptosis, we hypothesized that miRNAs are involved in regulating the onset of ferroptosis by targeting GPX4. Through database analysis and experimental validation, we selected miR-135b-3p as a key target of our study and confirmed through *in vitro* and *in vivo* experiments that miR-135b-3p could promote ferroptosis by inhibiting GPX4 expression in myocardial I/R injury.

## Materials and Methods

### Animal Model

Male Sprague–Dawley rats aged 8–10 weeks and weighing 220 g were obtained from the Nanjing Biomedical Research Institute of Nanjing University. All animal experiments complied with the Animal Research: Reporting *in vivo* Experiments (ARRIVE) guidelines (Supplementary Table 1. The ARRIVE guidelines 2.0 author checklist). The protocol was approved by the Ethics Committee of the Affiliated Hospital of the Nanjing University of Chinese Medicine. Following acclimatization for 1 week, the rats were divided into five groups of six rats each before the experiment. The establishment of the myocardial I/R model was based on previous studies (14). Sodium pentobarbital (45 mg/kg, i.p.) was used to anesthetize the rats, and the left coronary artery (LCA) was exposed using left thoracotomy at the fifth intercostal space. Following the LCA ligation with 7-0 silk sutures, a smooth catheter was applied to the artery to achieve ischemia for 30 min. The rats were then sacrificed 120 min after reperfusion. Rats in the sham group (without the LCA I/R) underwent surgery and were treated with saline. The miR-135b-3p group rats were injected with miR-135b-3p overexpression virus or knockdown lentivirus (1 × 10^8^ U/ml, 0.2 ml), respectively, for five consecutive days before surgery. The detailed animal grouping information in this study is listed in Supplementary Figure 1.

### Cell Culture and Establishment of the Hypoxia/Reoxygenation (H/R) Model

The rat myocardial cell line H9C2 was purchased from the Cell Resource Center of the Shanghai Academy of Sciences. H9C2 cells were cultured in DMEM supplemented with 10% fetal bovine serum and 100 units/ml of penicillin-streptomycin (MP Biomedicals). The cells were maintained in a humidified incubator containing 5% CO_2_ at 37°C. When the cells reached 80% confluence, the DMEM was replaced with serum-free and sugar-free medium. The cells were then placed in a 37°C hypoxia incubator containing 95% N_2_ and 5% CO_2_ for 6 h (17, 18). After hypoxia, the medium was replaced with a fresh medium and refilled with air containing 5% CO_2_ to establish I/R injury in cells.

### Cell Treatment and Cell Transfection

H9C2 cells cultured in 100-mm plastic dishes were allowed to adhere to the plate at 37°C in 5% CO_2_ for 6 h. Subsequently, cells were treated with 50 μM ferroptosis activator Erastin (MCE, China) or 1 μM ferroptosis inhibitor ferrostatin-1 (Fer-1, MCE, China) and incubated for 24 h. The cells were seeded in six-well plates at 1.0 × 10^5^/ml. When the confluency of cells reached 60%, transfection was performed. The GPX4 overexpression plasmid, miR-135b-3p mimics, and inhibitor were purchased from Synthgene (Nanjing, China), and the transfection was performed using Lipofectamine 2000 (Thermo-Scientific, USA) according to the manufacturer's instructions.

### ELISA

Rat blood was collected after reperfusion. After centrifugation at 3,000 rpm for 10 min at 4°C, 100 μl of serum was obtained. The activities of specific marker enzymes, including creatine phosphokinase (CK), lactic dehydrogenase (LDH), and cardiac troponin T (cTnT), were assessed according to the manufacturer's instructions (R&D Systems).

### Iron Assay

Intracellular ferrous iron (Fe^2+^) levels in rat myocardial tissues or H9C2 cells were measured using an iron assay kit (Abcam, USA) according to the manufacturer's instructions. Briefly, samples were collected and washed in cold PBS and then homogenized in 5X volumes of iron assay buffer on ice. The supernatant was collected, an iron reducer was added to each sample before mixing, and the samples were incubated at 25°C for 30 min. Thereafter, an iron probe was added to each sample before mixing and incubating at 25°C for 60 min. The output was measured immediately using a colorimetric microplate reader (optical density [OD], 593 nm).

### Analysis of Cell Viability

Cells were seeded in 96-well plates at a density of 2 × 10^3^ cells/well (200 μl/well) and cultured for 24 h. Subsequently, 5 mg/ml of the 3-(4,5-dimethyl-2-thiazolyl)-2,5-diphenyl-2H-tetrazolium bromide (MTT) reagent (Sigma, USA) was added (20 μl/well) and incubated at 37°C for another 4 h. Thereafter, the medium was removed and replaced with 150 μl/well DMSO (Sigma, USA), followed by vigorous shaking at room temperature for 10 min to solubilize the dark blue formazan crystals formed. Cell viability was evaluated by measuring the optical absorbance at 490 nm (OD490) using an Elx800 enzyme immunoassay analyzer (Bio-TEK, USA). The cell viability index was calculated as the experimental OD value/control OD value.

### RNA Isolation and Reverse Transcription-Quantitative PCR

Total RNA was isolated from the myocardial tissues or cultured cells using TRIzol® reagent (Thermo-Scientific, USA) and RNA was reverse transcribed to cDNA from 1 μg of total RNA using a PrimeScript RT reagent kit with gDNA Eraser (Takara, Japan) according to the manufacturer's protocol. The reaction conditions were as follows: 42°C for 2 min, 37°C for 15 min, and 85°C for 5 s. RT-PCR was performed using the SYBR green PCR kit on an Applied Biosystems 7300 sequence detection system (Applied Biosystems, USA). *U6* levels were used to normalize the relative abundance of miR-135b-3p, and *Gapdh* was used to normalize the expression of *Gpx4*, ferritin heavy chain 1 (*Fth1*), *Ascl4*, nicotinamide adenine dinucleotide phosphate oxidase 1 (*Nox1*), and cyclooxygenase 2 (*Cox2*). RT-qPCR reactions were performed in a 96-well plate at 95°C for 10 min, followed by 40 cycles of 95°C for 15 s, and 60°C for 60 s, according to the manufacturer's specifications. The primers used in this study are listed in Supplementary Table 2.

### Western Blotting Analysis

Total protein from H9C2 cells or myocardial tissues was extracted using RIPA lysis buffer. Proteins in the samples (20 μg) were separated *via* 10% SDS-PAGE and then transferred to a polyvinylidene difluoride membrane (Millipore, USA). Membranes were then incubated with 5% non-fat milk containing 0.1% PBST for 2 h at room temperature to block nonspecific binding and incubated with primary antibodies against GPX4 (1:1,000, Abcam), FTH1 (1:1,000, Abcam), ACSL4 (1:5,000, Abcam), NOX1 (1:5,000, Abcam), COX1 (1:500, Abcam), and GAPDH (1:1,000, Abcam) at 4°C overnight, followed by incubation with goat anti-rabbit HRP-conjugated secondary antibody (1:5,000, Abcam) at room temperature for 2 h. Protein bands were visualized using an enhanced chemiluminescence kit (Synthgene, China), and GAPDH was used as an internal control for the relative protein expression. Protein bands were quantified using the ImageJ software.

### Luciferase Reporter Assay

The entire 3′-UTR of *Gpx4* containing the predicted binding sites for miR-135b-3p was amplified and inserted into a luciferase reporter plasmid (Synthgene, China). To assess the binding specificity, the sequences that interacted with miR-135b-3p were mutated, and the mutant *Gpx4* 3′-UTR was inserted into an equivalent luciferase reporter plasmid. For the luciferase reporter assay, cells were plated in 24-well plates, and each well was transfected with 1 μg of luciferase reporter plasmid, 1 μg of β-galactosidase plasmid (internal control), and 100 pmol of miR-135b-3p mimic or control mimic using Lipofectamine 2000 (Thermo Fisher Scientific, USA). After 48 h, luciferase signals were measured using a luciferase assay kit according to the manufacturer's protocol (Promega Corporation, USA) (19, 20).

### Hematoxylin and Eosin Staining

After excising the myocardial tissue from rats, the tissues were fixed with 4% paraformaldehyde for 24 h and paraffin embedded. Sections (4 μm) were cut and stained with HE (21).

### The 2,3,5-Triphenyltetrazolium Chloride Staining Assay

At the end of 120 min of reperfusion, the hearts were collected and frozen at −20°C. Frozen hearts were cut into 1-mm sections that were incubated in 1% TTC solution at 37°C for 10 min and then fixed in 4% paraformaldehyde for 24 h. The sections were photographed using a digital camera. The infarcted areas were not stained by TTC (22, 23). The infarcted (unstained) and non-infarcted (stained) areas were measured in each section using ImageJ by a blinded investigator.

### Lipid Peroxidation Assay

Cells were treated as indicated, trypsinized, and resuspended in a medium supplemented with 10% FBS. A 10 μM solution of C11-BODIPY (Thermo Fisher, USA) was added, and samples were incubated for 30 min at 37°C with 5% CO_2_ and protection from light. The cells were washed twice with PBS to remove excess C11-BODIPY. The fluorescence of C11-BODIPY was measured using a fluorescence microscope (Nikon, Japan) (24).

### Echocardiographic Measurements

Cardiac function and structure were assessed using the MyLab™Eight Platform (Esaote, Italy). Briefly, rats were anesthetized with isoflurane (5%) using ventilation equipment, fur was carefully removed from the left chest, and two-dimensional echocardiographic measurements were obtained. Left ventricular internal diastolic and systolic diameter (LVIDd and LVIDs, respectively) and the left ventricular ejection fraction and fractional shortening (LVEF and LVFS, respectively) were measured using M-mode tracing.

### Statistical Analysis

All data are presented as the mean ± standard deviation (SD) of three independent experiments. One-way ANOVA and Duncan's multiple range tests were used to evaluate the mean differences between groups and within groups. Statistical significance was set at *p* < 0.05.

## Results

### Ferroptosis Involved in the Process of Rat Myocardial I/R

**Figure 1:**
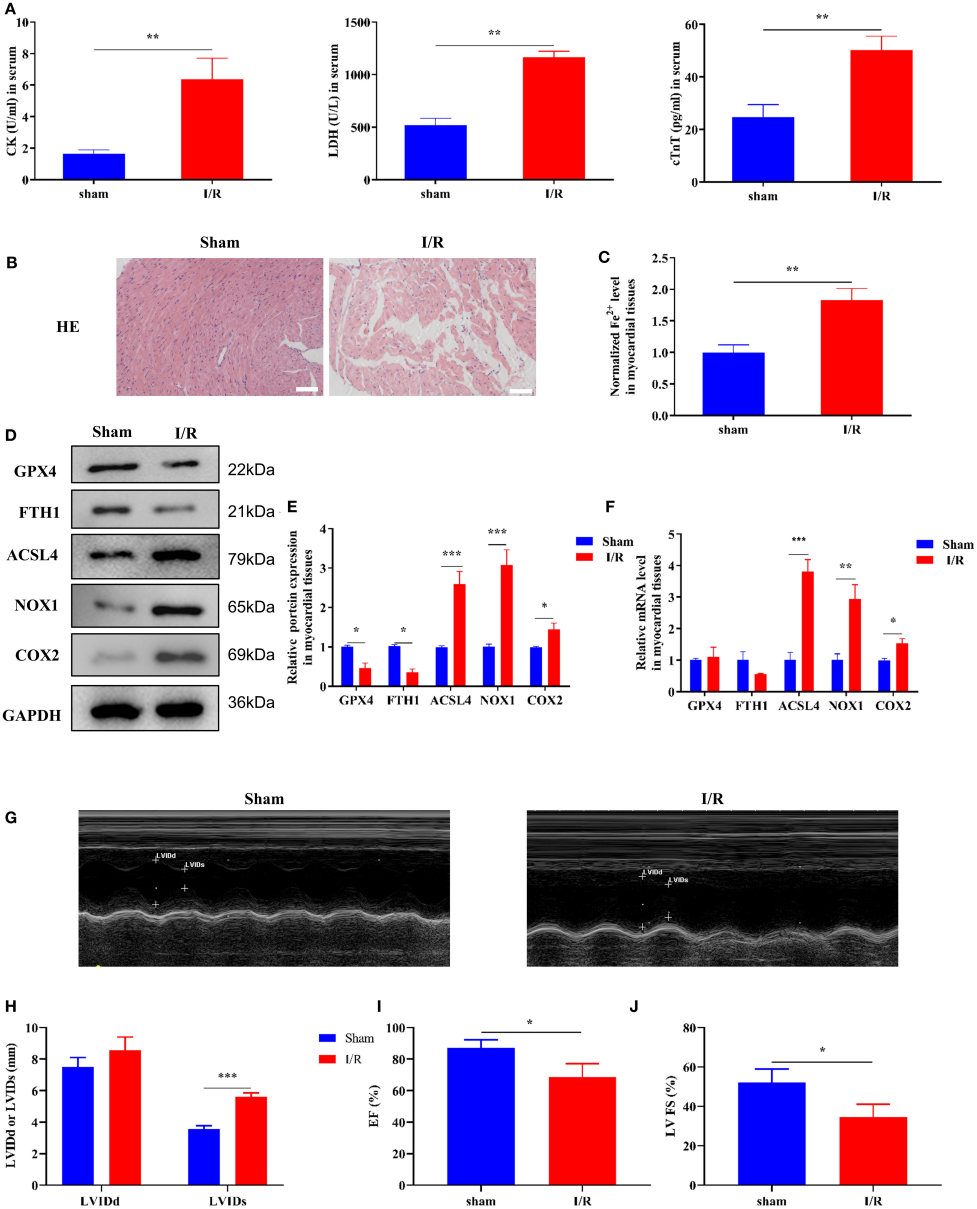
Ferroptosis occurs in myocardial I/R rats. This part of the study was based on sham and I/R rats (*n* = 6). **(A)** The concentration of CK, LDH, and cTnT in serum was determined using ELISA. **(B)** HE staining was performed to detect myocardial tissue injury. Scale bar = 50 μm. **(C)** Iron assay kit was used to measure the change in ferrous iron levels in the myocardial tissues of sham and I/R rats. Scale bar = 400 μm. **(D,E)** Western blotting was used to detect the levels of GPX4, FTH1, NOX1, ASCL4, and COX2 in sham and I/R rat myocardial tissues; quantitative analysis of the protein levels is shown in E. **(F)** RT-qPCR analysis was used for determining the GPX4, FTH1, NOX1, ASCL4, and COX2 mRNA levels. **(G–J)** LV short-axis view by transesophageal echocardiography in M-mode. ***p < 0.001, **p < 0.01, *p < 0.05. * denotes the difference of the I/R group compared with the sham group. Error bars represent the mean ± SD of the experiments in triplicates.

Initially, we established a myocardial I/R injury model in rats and collected myocardial tissue and serum samples. We performed HE staining and ELISA to detect tissue structure and cardiac injury marker expression, respectively. The serum CK, LDH, and cTnT levels were significantly increased in the I/R group compared to those in the sham group (Figure 1A). As shown in Figure 1B, the myocardial cells were well-ordered with regular structure and myocardial fibers were intact in the sham group, but myocardial cells were disordered and a few myocardial fibers were broken in the I/R group. Results of the analysis of the cardiac injury biomarker expression and HE staining revealed significant damage in the hearts of the rats in the I/R group. To verify the involvement of ferroptosis in the process of cardiac injury, we used the iron assay kit to measure ferrous iron levels in myocardial tissues, and the results showed that in the I/R group, the normalized Fe^2+^ levels were elevated (Figure 1C). The ferroptosis-related gene expression results are shown in Figures 1D–F. The protein and mRNA expression of GPX4, FTH1, ACSL4, NOX1, and COX2 was detected through Western blotting and RT-qPCR, respectively. ACSL4, NOX1, and COX2 mRNA and protein levels were significantly higher in the I/R group than in the sham group. However, we found that GPX4 and FTH1 were significantly reduced at the protein level in the I/R group but not at the mRNA level, suggesting the presence of factors inhibiting the translation of these two genes.

To visualize the cardiac damage in the I/R group, we performed transthoracic echocardiography and M-mode tracings in rats in the I/R group. The results showed that LVIDs were significantly higher while LVFS and LVEF% were significantly lower in the I/R group than those in the sham group. In addition, the mean LVIDd values were higher in the I/R group than the sham group, and there were no statistically significant differences between the two groups (Figures 1G–J). The above results showed that the heart function of rats was disrupted after I/R injury.

### miR-135b-3p Targets *Gpx4*, and Its Expression Increases in Myocardial I/R Rats

**Figure 2:**
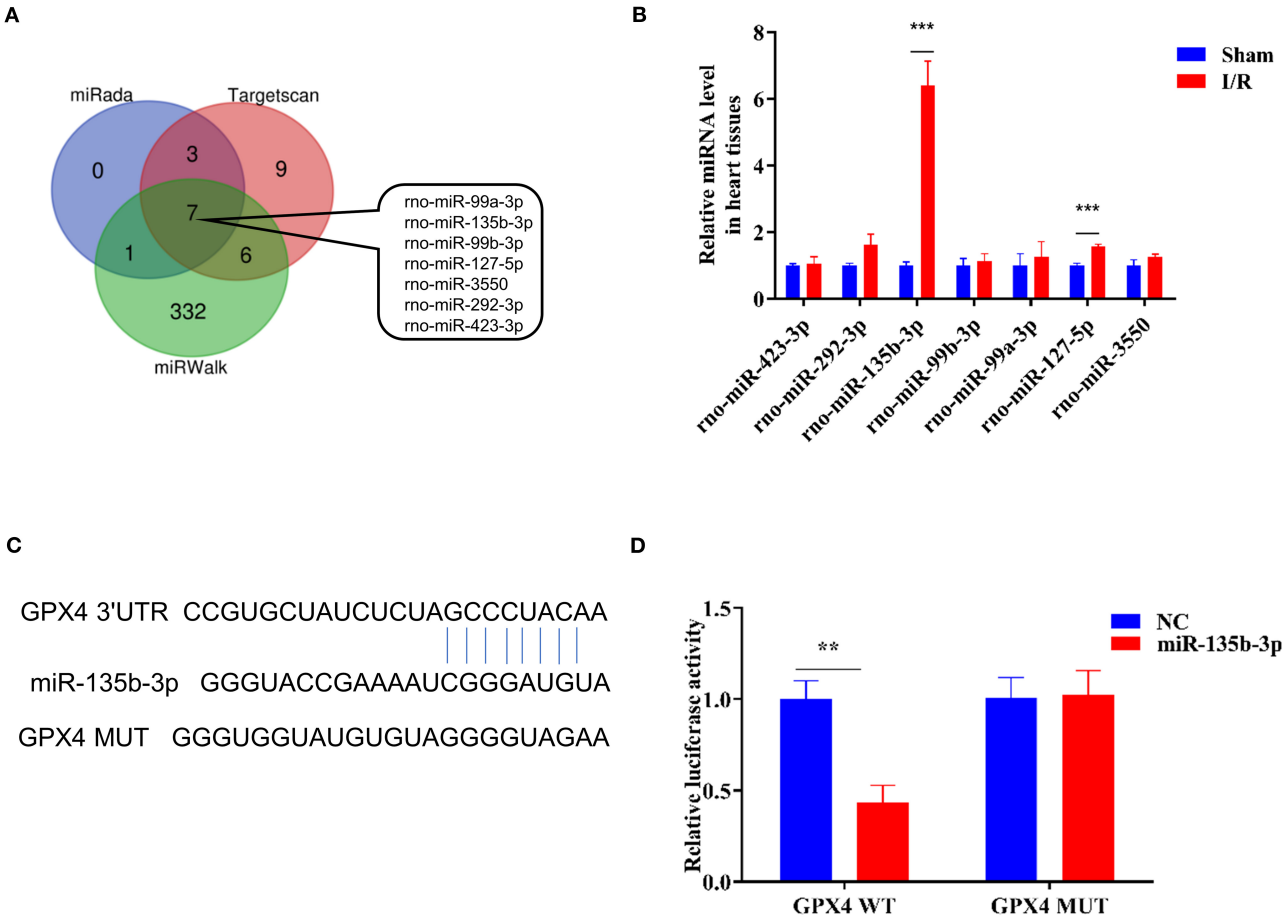
*Gpx4* is a direct target of miR-135b-3p. **(A)** Prediction of miRNAs that target *Gpx4* using TargetScan, miRWalk, and miRada database. **(B)** Detection of miRNA expression in sham and I/R myocardial tissues of rats using RT-qPCR (*n* = 3). **(C)** The putative miR-135b-3p-binding sites in 3'UTR of wild-type and mutant *Gpx4*. **(D)** Relative luciferase activity of the cells co-transfected with constructed luciferase reporters (*Gpx4*-3′UTR-WT and *Gpx4*-3′UTR-MUT), pRL-TK vectors, and miR-135b-3p mimics or negative control (*n* = 3). ***p < 0.001, **p < 0.01. Error bars represent the mean ± SD of triplicate experiments.

To explore the miRNA-mediated regulation of *Gpx4* expression, we used TargetScan, miRWalk, and miRada to predict and screen the miRNAs targeting *Gpx4* (Supplementary Dataset 1), and the results were presented in the form of a Venn diagram (Figure 2A), which was drawn using the tool available from http://bioinformatics.psb.ugent.be/webtools/Venn/. The expression of miRNAs found by database analysis, in the sham and I/R groups, was examined using RT-qPCR. We found that miR-135b-3p expression was significantly increased in myocardial I/R (Figure 2B). The prediction results of the TargetScan Release 7.0 database showed that the 3′-UTR of *Gpx4* mRNA possesses putative binding sites for miR-135b-3p (Figure 2C). To further confirm the direct binding of miR-135b-3p to the *Gpx4* mRNA 3′UTR, a luciferase activity assay was performed. H9C2 cells were co-transfected with *Gpx4-*3′UTR-WT, *Gpx4*-3′UTR-MUT, miR-135b-3p, or negative control mimics. miR-135b-3p significantly inhibited the luciferase activity of *Gpx4*-3′UTR-WT, whereas that of *Gpx4*-3′UTR-MUT was not decreased (Figure 2D), suggesting that miR-135b-3p was able to suppress the translation by binding to the 3′UTR of *Gpx4* mRNA. These results demonstrate that the miR-135b-3p expression increases in myocardial I/R tissue and directly regulates the expression of *Gpx4* in H9C2 cells.

### Ferroptosis Occurs in H9C2 Cells After H/R and Is Accompanied by Altered Expression of miR-135b-3p

**Figure 3:**
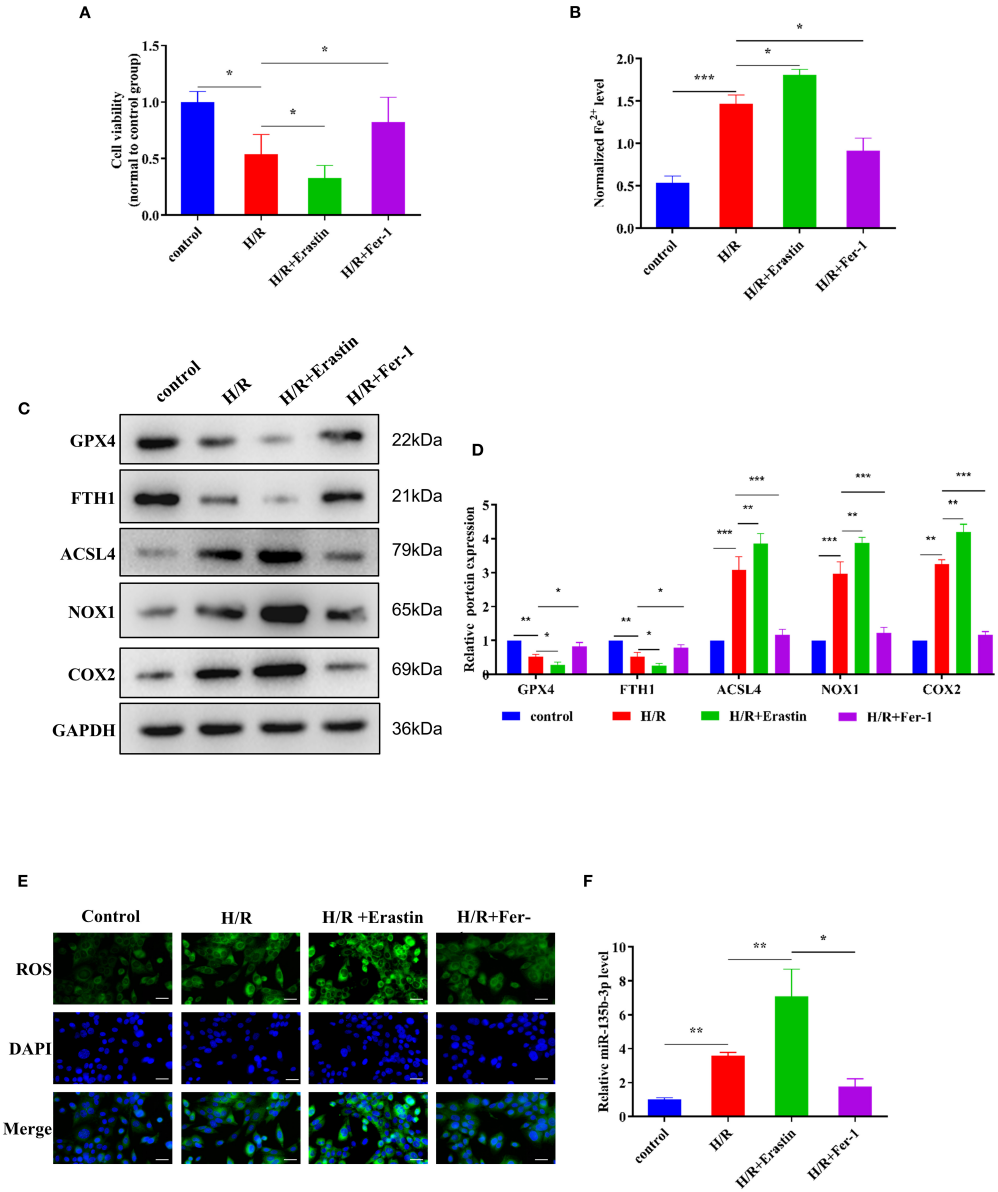
Ferroptosis affects cell survival and miR-135b-3p expression in H9C2 cells after H/R. This part of the study was based on the untreated H9C2 cells and H/R-treated H9C2 cells that were treated with erastin or Fer-1 (*n* = 3). **(A)** MTT assay was conducted to detect the effect of erastin and Fer-1 on the viability of H/R H9C2 cells. **(B)** The ferrous iron level in H9C2 cells with different treatments was measured by using the iron assay kit. **(C)** Western blotting was used for detecting GPX4, FTH1, NOX1, ASCL4, and COX2 expression in different H9C2 cells; quantitative analysis is shown in **(D)**. **(E)** C11-BODIPY was used to detect the ROS level in H9C2 cells subjected to different treatments. DAPI staining was used for nuclear localization. Scale bar = 50 μm. **(F)** RT-qPCR analysis of the expression of miR-135b-3p in H9C2 cells. ***p < 0.001, **p < 0.01, *p < 0.05. Error bars represent the mean ± SD of triplicate experiments.

Erastin is an oncogenic RAS-selective lethal small molecule that triggers a unique iron-dependent form of non-apoptotic cell death in various cell types. Fer-1 inhibits the accumulation of cytoplasmic and lipid ROS induced by erastin (25). To further explore the roles of ferroptosis in myocardial I/R, we cultured rat myocardial H9C2 cells under H/R conditions to induce I/R damage. Cell viability was reduced in the H/R group compared to the control group, and treatment with erastin further reduced cell viability, whereas treatment with Fer-1 restored the viability of H9C2 cells subjected to H/R (Figure 3A). These results confirmed that H/R treatment could promote ferroptosis in cells. An iron assay kit was used to measure ferrous iron levels in H9C2 cells. The results showed that the normalized Fe^2+^ level in the H/R group was significantly higher than that in the control group. The normalized Fe^2+^ level in H/R cells was increased after treatment with erastin but decreased after Fer-1 treatment (Figure 3B). Subsequently, we examined the expression of ferroptosis-associated proteins by Western blotting. GPX4 and FTH1 expression decreased and ACSL4, NOX1, and COX2 expression increased in H/R cells compared to the control cells. When H/R cells were treated with erastin, GPX4 and FTH1 expression was inhibited, while ACSL4, NOX1, and COX2 expression increased. As shown in Figures 3C,D, the ferroptosis-related gene expression in Fer-1-treated cells was completely contrary to that in the erastin-treated cells. Immunofluorescence results showed that ROS levels were upregulated in H/R cells and the erastin-treated H/R cells, whereas Fer-1 treatment downregulated the ROS levels in H/R cells (Figure 3E). These results suggest that ferroptosis is involved in myocardial H/R injury. Furthermore, we used RT-qPCR to detect changes in miR-135b-3p expression in different groups and found that miR-135b-3p expression was positively correlated with the severity of ferroptosis (Figure 3F). These results suggest that miR-135b-3p may be involved in myocardial cell ferroptosis.

### miR-135b-3p Promotes Cell Ferroptosis by Reducing GPX4 Expression in H9C2 Cells After H/R

**Figure 4:**
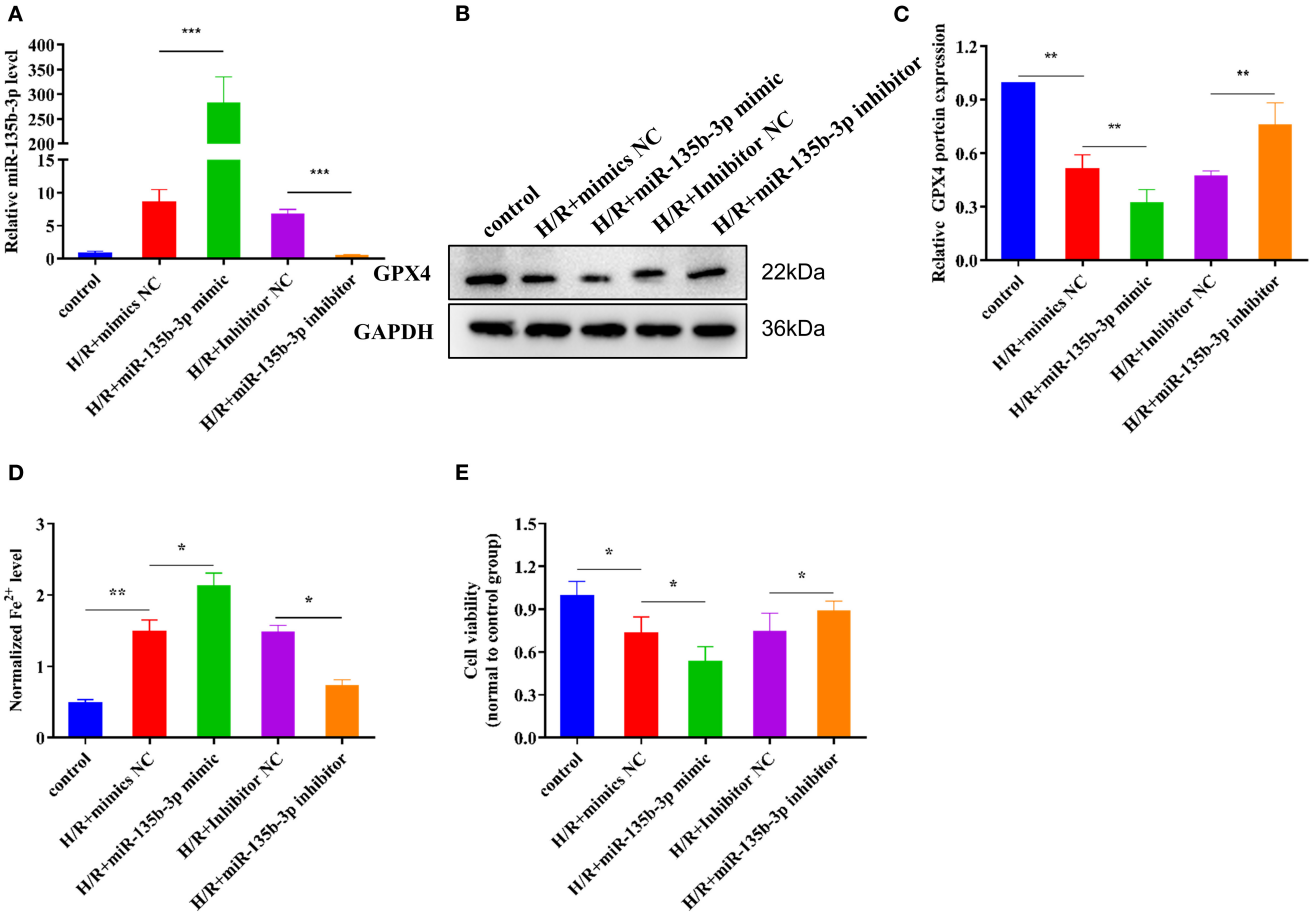
miR-135b-3p promotes ferroptosis in H9C2 cells after H/R. This part of the study was based on untreated H9C2 cells and H/R-treated H9C2 cells, where miR-135b-3p expression was altered by mimics or an inhibitor in H/R H9C2 cells (*n* = 3). **(A)** RT-qPCR-based detection of the expression of miR-135b-3p in control or H/R H9C2 cells after transfection with miR-135b-3p mimics, inhibitor, or corresponding control. **(B)** The expression of GPX4 in miR-135b-3p-overexpressing or miR-135b-3p-knockdown H/R H9C2 cells was detected by Western blotting; quantitative analysis is shown in **(C)**. **(D)** Ferrous iron level was detected in miR-135b-3p-overexpressing or miR-135b-3p-knockdown H/R H9C2 cells by using the iron assay kit. **(E)** MTT assay was used to analyze cell viability after altering the expression of miR-135b-3p. ***p < 0.001, **p < 0.01, *p < 0.05. Results represent the average of three independent experiments. Error bars represent the mean ± SD.

Previous studies have demonstrated that miR-135b-3p can target GPX4 and is aberrantly expressed in ferroptosis; therefore, we determined whether miR-135b-3p promotes ferroptosis by regulating GPX4 expression. To explore the miR-135b-3p effect in H/R-induced H9C2 cells, we first transfected miR-135b-3p mimics, negative control mimics, miR-135b-3p inhibitor, and negative control inhibitor in H/R-induced H2C9 cells. miR-135b-3p expression detected by RT-qPCR was used to confirm the transfection efficiency (Figure 4A). Western blotting results showed a significant negative correlation between miR-135b-3p and GPX4 (Figures 4B,C). miR-135b-3p increased the normalized Fe^2+^ levels (Figure 4D). The cell viability assay showed that miR-135b-3p mimics significantly decreased the cell viability compared to the negative control mimics group, whereas the viability of cells transfected with miR-135b-3p inhibitor was increased compared to the negative control group (Figure 4E). Based on these results, we concluded that miR-135b-3p regulated the expression of GPX4 and ferroptosis in H/R-induced H9C2 cells.

**Figure 5:**
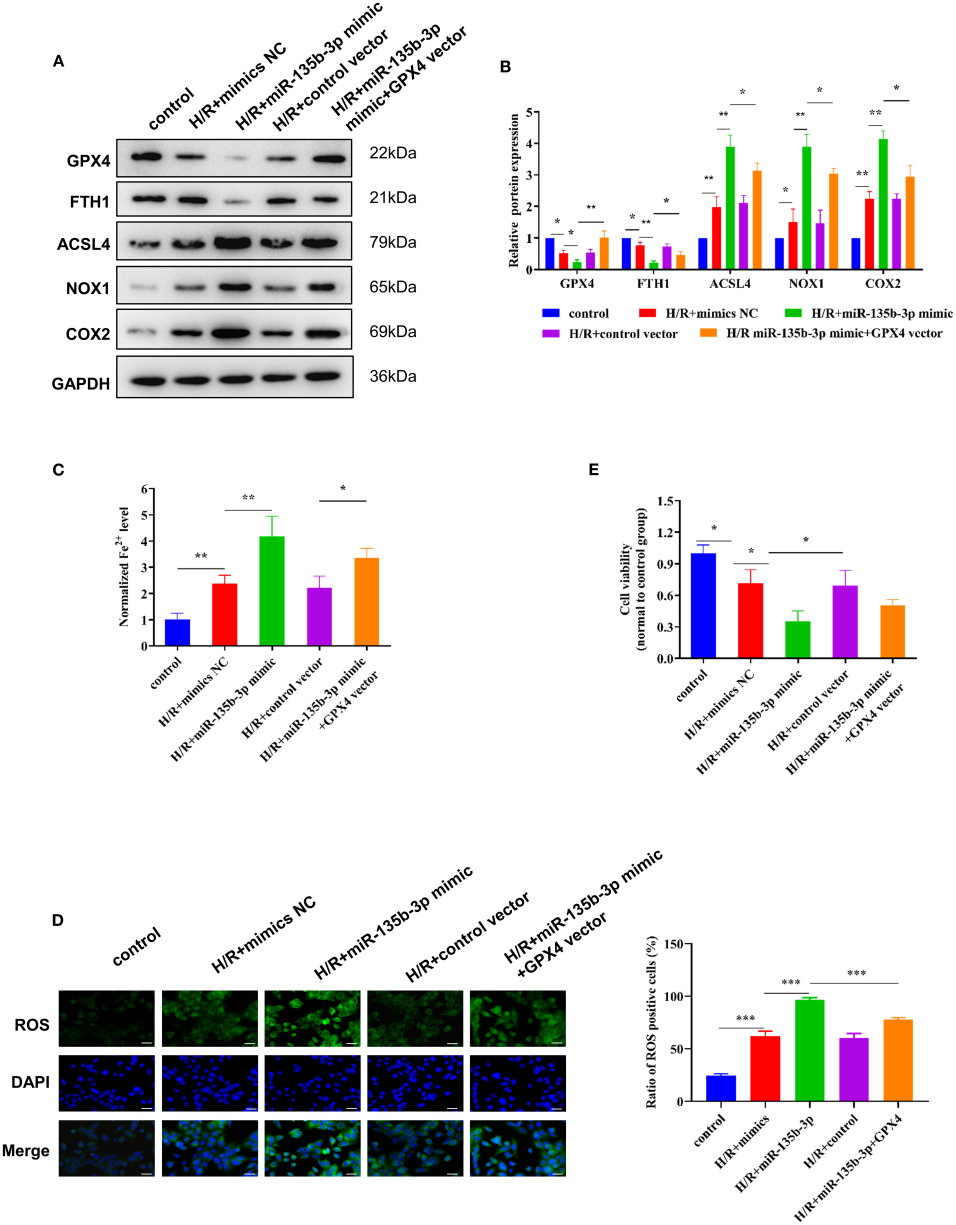
miR-135b-3p promotes ferroptosis through inhibition of GPX4 expression in H9C2 cells after H/R. This part of the study was based on untreated H9C2 cells and H/R-treated H9C2 cells, where miR-135b-3p and GPX4 expression was altered in H/R H9C2 cells (*n* = 3). **(A)** Western blots indicating the levels of GPX4 and other ferroptosis-related proteins in H/R H9C2 cells overexpressing miR-135b-3p or H/R H9C2 cells overexpressing miR-135b-3p and GPX4. The quantitative analysis is shown in **(B)**. **(C)** The ferrous iron concentration was measured in H/R H9C2 cells overexpressing miR-135b-3p or H/R H9C2 cells overexpressing miR-135b-3p and GPX4 by using the iron assay kit. **(D)** C11-BODIPY was used to detect the ROS levels in cells. DAPI staining was used for nuclear localization. Scale bar = 50 μm. The quantitative analysis is shown on the right. **(E)** MTT assay was used to evaluate the viability of H/R H9C2 cells overexpressing miR-135b-3p or H/R H9C2 cells overexpressing miR-135b-3p and GPX4. ***p < 0.001, **p < 0.01, *p < 0.05. Results represent the average of three independent experiments; error bars represent the mean ± SD.

To further explore the influence of miR-135b-3p on ferroptosis through GPX4, we used the GPX4 plasmid to increase the expression of GPX4, which was confirmed by Western blotting. The Western blot results also showed that the increased expression of GPX4 and miR-135b-3p leads to increased expression of FTH1 and decreased expression of ACSL4, NOX1, and COX2 in H/R myocardial cells, compared to the cells transfected only with miR-135b-3p mimics (Figures 5A,B). Results from the assays for normalized Fe^2+^ levels, cell viability, and ROS levels confirmed that restoration of GPX4 expression could reduce cell ferroptosis and increase cell viability (Figures 5C–E). These data suggest that miR-135b-3p promotes cell ferroptosis by inhibiting GPX4 expression in H/R myocardial cells.

### miR-135b-3p/GPX4 Promotes Cell Ferroptosis and Aggravates Myocardial I/R Injury *In vivo*

**Figure 6:**
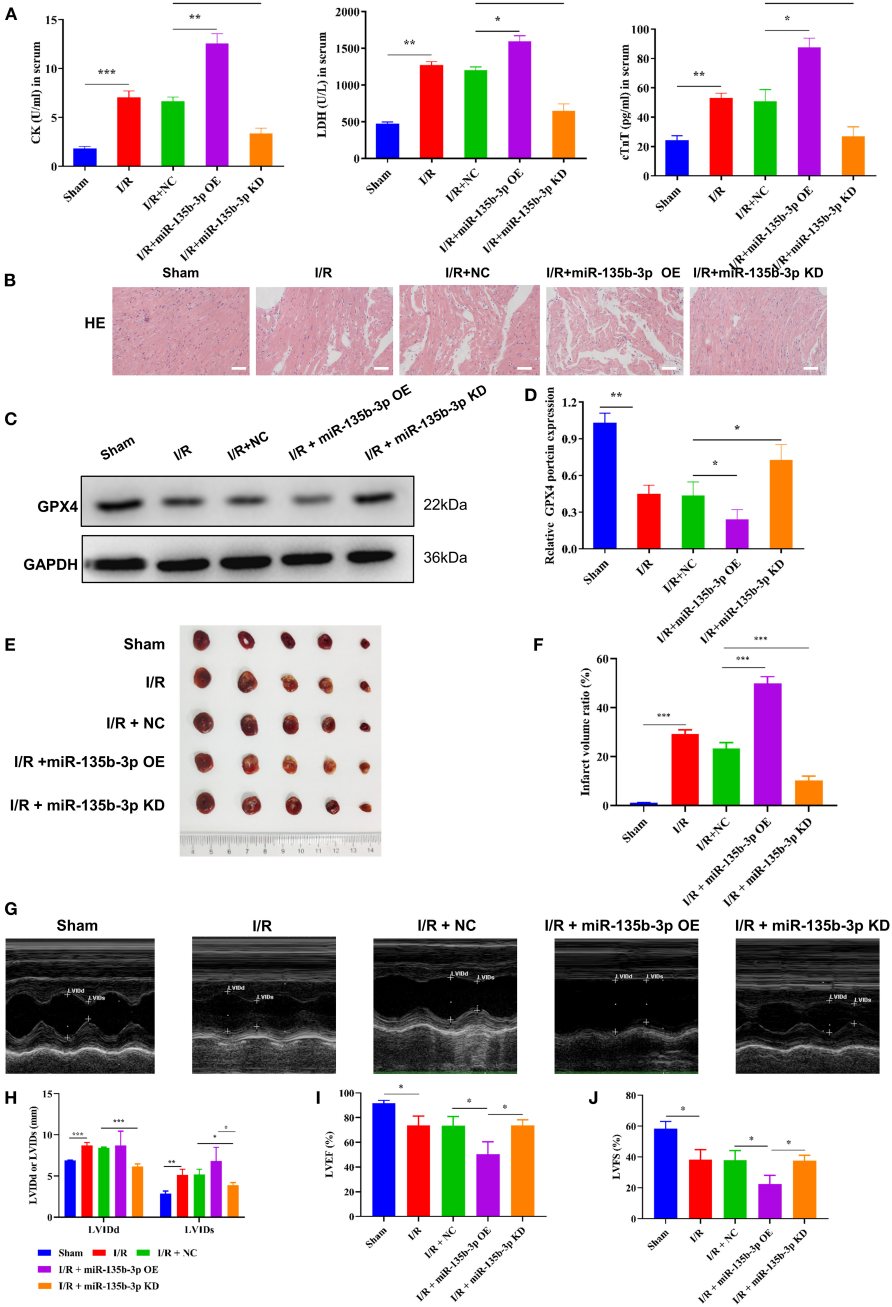
miR-135b-3p enhances ferroptosis by reducing GPX4 expression *in vivo*, ultimately exacerbating myocardial I/R injury. This part of the study was based on sham and I/R rats, where miR expression in I/R rats was regulated by lentivirus injection (*n* = 6). **(A)** The expression of CK, LDH, and cTnT in serum was detected using ELISA. **(B)** Histopathology was analyzed using HE staining. Scale bar = 400 μm. **(C,D)** Western blotting was used to detect the levels of GPX4 in myocardial tissues of rats, and the quantitative analysis is shown in **(D)**. **(E,F)** Infarct volumes were evaluated by TTC staining of hearts; quantitative analysis is shown in **(F)**. **(G–J)** LV short-axis view by transesophageal echocardiography in M-mode. ***p < 0.001, **p < 0.01, *p < 0.05. Results represent the average of three independent experiments; error bars represent the mean ± SD.

To confirm that miR-135b-3p promotes cell ferroptosis by suppressing GPX4 expression *in vivo*, we constructed an I/R rat model by injecting the control virus, miR-135b-3p overexpression virus, or knockdown virus into the tail vein. After reperfusion, the myocardial tissue and serum were collected. ELISA results showed a positive correlation between miR-135b-3p and CK, LDH, and cTnT levels, indicating that miR-135b-3p could aggravate myocardial I/R injury (Figure 6A). HE staining showed that overexpression of miR-135b-3p could lead to myocardial cell disorder and a rise in cell death, and knockdown of miR-135b-3p could restore these effects (Figure 6B). Western blot results showed that GPX4 expression was reduced in the I/R group, but inhibiting miR-135b-3p expression restored the GPX4 expression (Figures 6C,D).

To visually assess the infarct volume, we performed TTC staining, which showed that the hearts of rats in the I/R group showed distinct areas of infarction compared to the sham group and that the infarction became more severe after miR-135b-3p overexpression (Figures 6E,F). In addition, we performed transthoracic echocardiography and M-mode tracing in rats from five groups. The results showed that LVIDd and LVIDs values were significantly higher, whereas LVFS% and LVEF% were significantly lower, in the I/R group than in the sham group, as demonstrated in Figure 1. In addition, we found that miR-135b-3p expression had a significant effect on these four indicators: miR-135b-3p overexpression led to an increase in LVIDd and LVIDs and a decrease in LVFS% and LVEF% in rats (Figures 6G–J).

Taken together, the *in vivo* experiments confirmed that miR-135b-3p promotes cellular ferroptosis by downregulating GPX4 expression, thereby exacerbating myocardial I/R injury.

## Discussion

Myocardial I/R can lead to a large amount of Fe^2+^ influx and ROS production, which is the main mechanism of pathogenesis of cell damage (26). Myocardial I/R is related to the occurrence and development of various clinical diseases, such as myocardial infarction and atherosclerosis, and affects patient recovery (16–18, 27). Therefore, exploring the mechanisms of myocardial I/R injury and its alleviation are of great significance for treating these diseases. In our study, we constructed a myocardial I/R rat model and detected the CK, LDH, and cTnT expression levels in the serum. An increase in the CK, LDH, and cTnT levels is indicative of myocardial I/R injury during myocardial perfusion (19, 20). Our results showed that CK, LDH, and cTnT expression was upregulated in the I/R model group, indicating a successful myocardial I/R model. Induction of ferroptosis has been reported in the myocardial tissue during I/R injury (21, 28). In the present study, we found that the level of Fe^2+^ increased during I/R injury, and the expression of ferroptosis-related genes changed significantly. Moreover, we found that the expression of GPX4 and FTH1 was downregulated at the protein level but there were no significant differences in their mRNA levels, suggesting that the reduced expression of transcription factors or epigenetic modifications may be involved in the inhibition of GPX4 and FTH1 translation. Classical ferroptosis is regulated by GPX4 signaling (5, 6); therefore, we hypothesized that miRNAs involved in regulating GPX4 translation might affect ferroptosis.

Many studies have reported that miRNAs regulate ferroptosis in cancers and other diseases (13, 22, 29). In gastric cancer cells, CAFs secrete exosome miR-522 to inhibit ferroptosis by targeting ALOX15 and blocking lipid-ROS accumulation (23). In upper gastrointestinal cancer, inhibition of AURKA or reconstitution of miR-4715-3p was shown to inhibit GPX4 expression and induce cell ferroptosis; this phenomenon represents a novel epigenetic mechanism mediating miR-4715-3p silencing and AURKA induction (24). However, in myocardial I/R injury, the effects of miRNAs on ferroptosis have not been reported. Nonetheless, miR-21 overexpression in the heart has been reported to reduce cardiomyocyte apoptosis and myocardial infarct size (30). Therefore, we speculated that miRNAs are also involved in myocardial I/R injury. As mentioned earlier, GPX4 plays a crucial role in the occurrence of ferroptosis, which is a key cause of myocardial injury caused by I/R. To investigate *GPX4* expression during myocardial I/R and the functional roles miRNAs may play in the process, we focused on the *GPX4*-targeting miRNAs and found that miR-135b-3p directly targets *GPX4* in I/R tissues. We transfected miR-135b-3p mimics or inhibitors into cultured cardiomyocytes to explore the roles of miR-135b-3p in the H/R model. We determined ferrous iron levels, cell viability, and *Gpx4* expression in the cells and found that transfection of miR-135b-3p mimics increased the ferrous iron levels and decreased *Gpx4 e*xpression and cell viability in cardiomyocytes after H/R. In contrast, transfection with miR-135b-3p inhibitor reduced the ferrous iron levels and increased *Gpx4* expression and cell viability in cardiomyocytes after H/R. These results suggest that miR-135b-3p promotes cell death in an iron-dependent manner *in vitro*. We further found that upregulation of *Gpx4* expression restored cell viability, ferrous iron levels, and lipid ROS levels compared with transfection of miR-135b-3p separately. These results confirm that miR-135b-3p affects ferroptosis in cardiomyocytes after H/R by regulating GPX4 expression. We then confirmed the effect of miR-135b-3p on ferroptosis by injecting a virus to upregulate or downregulate the expression of miR-135b-3p in rats. In addition to miR-135b-3p, we believe that other miRNAs might be involved in the regulation of I/R-related ferroptosis. Further exploration and studies are required to provide adequate experimental evidence for alleviating or avoiding I/R damage in clinical settings by targeting miRNAs.

In summary, we demonstrated the upregulation of miR-135b-3p in myocardial I/R rat cardiac muscle tissues and H/R myocardial cells. Moreover, increased expression of miR-135b-3p worsened the cell injury by promoting ferroptosis through inhibition of GPX4 expression. Therefore, targeting miR-135b-3p may be a potential therapeutic approach for myocardial I/R injury.

## Data Availability Statement

The original contributions presented in the study are included in the article/Supplementary Material, further inquiries can be directed to the corresponding author/s.

## Ethics Statement

The animal study was reviewed and approved by Ethics Committee of Affiliated Hospital of Nanjing University of Chinese Medicine.

## Author Contributions

XC: conceptualization and supervision. WS and PY: data curation and project administration. HS, PY, and XC: funding acquisition. RS, JG, and HW: investigation and resources. LS and PY: methodology. WS: software, visualization, and writing—original draft. HS: validation. WS, PY, and XC: writing—review and editing. All authors contributed to the article and approved the submitted version.

## Conflict of Interest

The authors declare that the research was conducted in the absence of any commercial or financial relationships that could be construed as a potential conflict of interest.

## Publisher's Note

All claims expressed in this article are solely those of the authors and do not necessarily represent those of their affiliated organizations, or those of the publisher, the editors and the reviewers. Any product that may be evaluated in this article, or claim that may be made by its manufacturer, is not guaranteed or endorsed by the publisher.

## Supplementary Material

Supplementary Table 1 The ARRIVE guidelines 2.0 author checklist.

Supplementary Table 2 The primer sequences of genes in RT-qPCR assay.

Supplementary Dataset 1 The result of TargetScan, miRWalk, and miRada to predict and screen the miRNAs targeting *Gpx4*.

Supplementary Figure 1 The detailed animal groupings information.

Supplementary Figure 2 The Full Scan WB images in the study.
