# Supplementary material for: miR-135b-3p Promotes Cardiomyocyte Ferroptosis by Targeting GPX4 and Aggravates Myocardial Ischemia/Reperfusion Injury
Source: Front Cardiovasc Med. 2021 Aug 13;8:663832. doi: 10.3389/fcvm.2021.663832 (PMC8414249; doi:10.3389/fcvm.2021.663832)
Supplement: Supplementary Table 2 — The primer sequences of genes in RT-qPCR assay. [file Table_2.docx]

**Table S2. Primer sequences of genes in RT-qPCR assay**

| **Gene** | **Forward Primer** | **Reversed Primer** |
| --- | --- | --- |
| *Gpx4* (rat) | 5’-AATCCTGGCCTTCCCTTGCA-3’ | 5’-GCCCTTGGGCTGGACTTTCA-3’ |
| *Fth1* (rat) | 5’-CCAGAACTACCACCAGGACTC-3’ | 5’-GTTTCTCAGCATGTTCCCTCT-3’ |
| *Acsl4* (rat) | 5’-AGCCGCACTGAAGAATTGTC-3’ | 5’-TCTACCCCCTTCTGTTGTGC-3’ |
| *Nox1* (rat) | 5’-TACGAAGTGGCTGTACTGGTTG-3’ | 5’-CTCCCAAAGGAGGTTTTCTG-3’ |
| *Cox2*（rat） | 5’-CCAGATGGCCAGAGGACTCA-3’ | 5’-TGTGAGTCCCGAGGGAATAGA-3’ |
| rno-miR-423-3p | 5’-GCAGTCGAGCCAGACTC-3’ | 5’-CCAGTTTTTTTTTTTTTTTGACTCC-3’ |
| rno-miR-292-3p | 5’-GCCGCCAGGTTTTGAG-3’ | 5’-GGTCCAGTTTTTTTTTTTTTTTACAC-3’ |
| rno-miR-135b-3p | 5’-TACATCCCGATTTTCGGTACCC-3’ | 5’-GTCCAGTTTTTTTTTTTTTTTGGGT-3’ |
| rno-miR-99b-3p | 5’-GGTTCGAGCAAAGATACCCA-3’ | 5’-GGTCCAGTTTTTTTTTTTTTTTGTC-3’ |
| rno-miR-99a-3p | 5’-GCAAGCTCGTTTCTATGGGT-3’ | 5’-GGTCCAGTTTTTTTTTTTTTTTCAG-3’ |
| rno-miR-127-5p | 5’-CTGAAGCTCAGAGGGCT-3’ | 5’-GGTCCAGTTTTTTTTTTTTTTTAATCAGA-3’ |
| rno-miR-3550 | 5’-AGCCGAGCCCATCC-3’ | 5’-CAGTTTTTTTTTTTTTTTCTAGGGCA-3’ |
