# Supplementary material for: miR-135b-3p Promotes Cardiomyocyte Ferroptosis by Targeting GPX4 and Aggravates Myocardial Ischemia/Reperfusion Injury
Source: Front Cardiovasc Med. 2021 Aug 13;8:663832. doi: 10.3389/fcvm.2021.663832 (PMC8414249; doi:10.3389/fcvm.2021.663832)
Supplement: Supplementary Figure 1 — The detailed animal groupings information. [file Image_1.pdf]

Supplementary FigureS1: The detailed animal groupings information

Animal Experiments Part I
（Figure1）

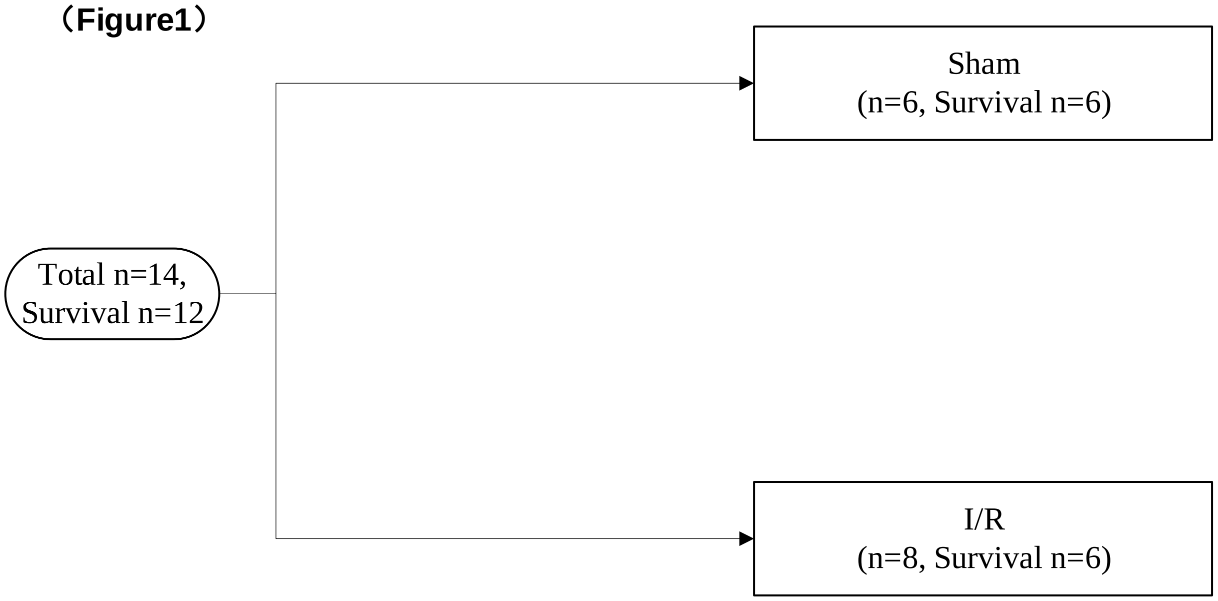

Animal Experiments Part 2
（Figure6）

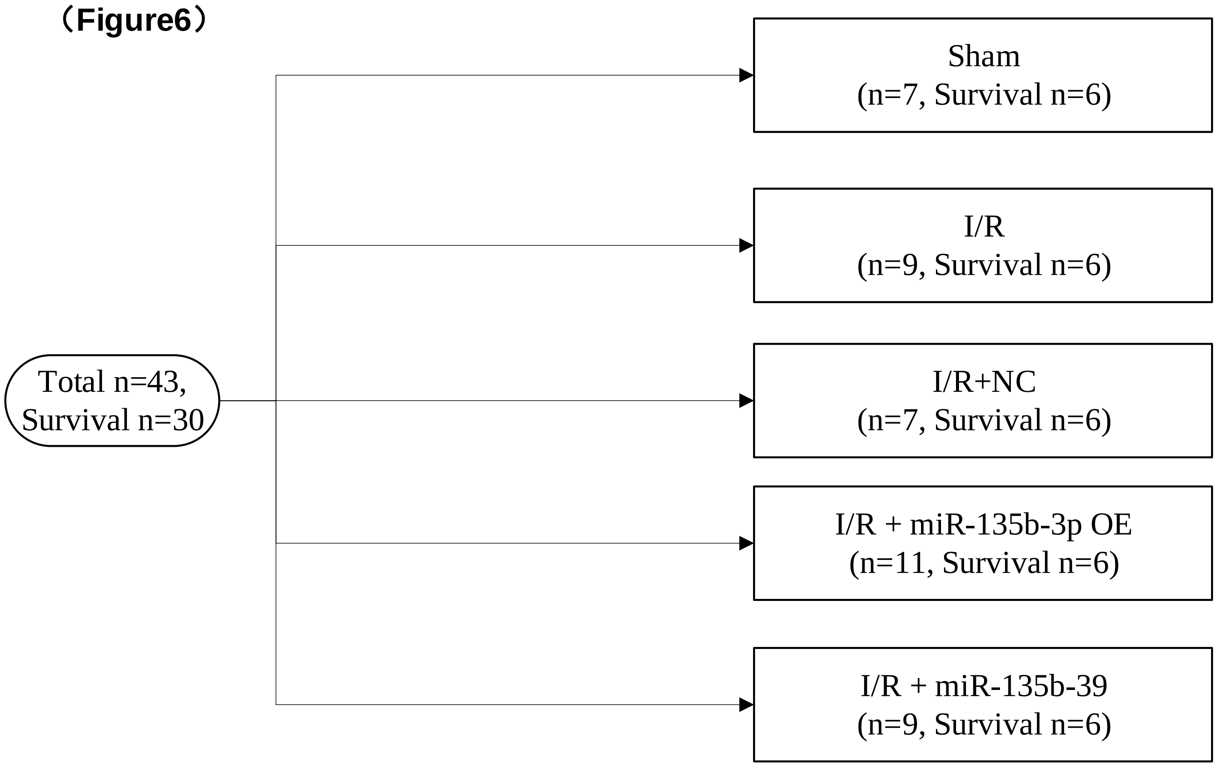

In order to end up with 6 rats in each group for the final study, we estimated the experimental animals based on a survival rate of approximately 20-30% for the rat molds and contacted the animal experimentation center managers to continue managing the rats that did not enter the experiment. If there are not enough surviving animals after surgery, they will continue to be replenished until there are 6 rats in each group.

Experiment part 1 prepared 18 rats (6×2÷0.7=17.1), and 14 rats actually entered the experiment. Experiment part 2 prepared 43 rats (6×5÷0.7=42.85), and 43 rats actually entered the experiment.
