# Supplementary material for: miR-135b-3p Promotes Cardiomyocyte Ferroptosis by Targeting GPX4 and Aggravates Myocardial Ischemia/Reperfusion Injury
Source: Front Cardiovasc Med. 2021 Aug 13;8:663832. doi: 10.3389/fcvm.2021.663832 (PMC8414249; doi:10.3389/fcvm.2021.663832)

Figure1D GPX4

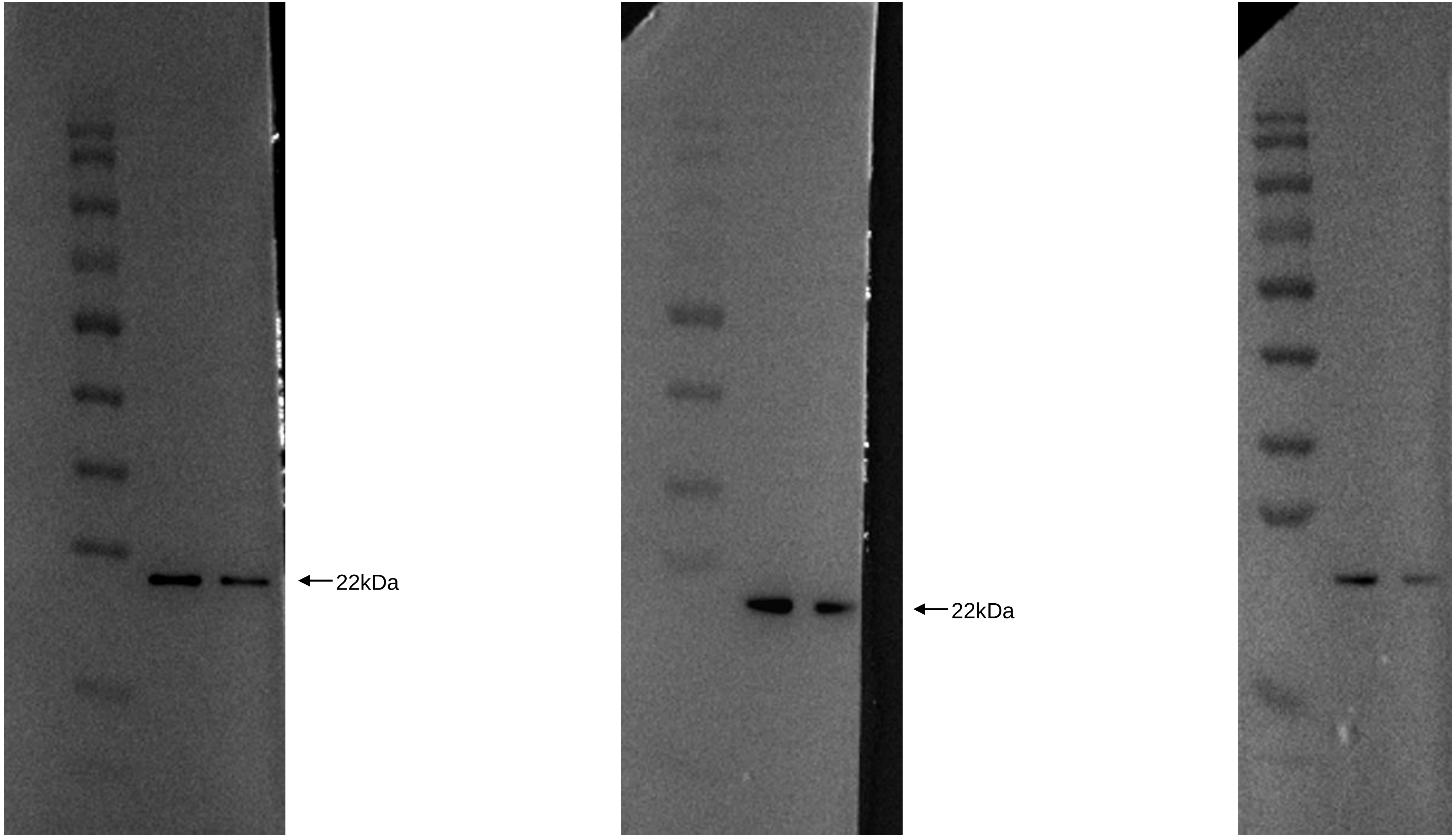

Figure1D FTH1

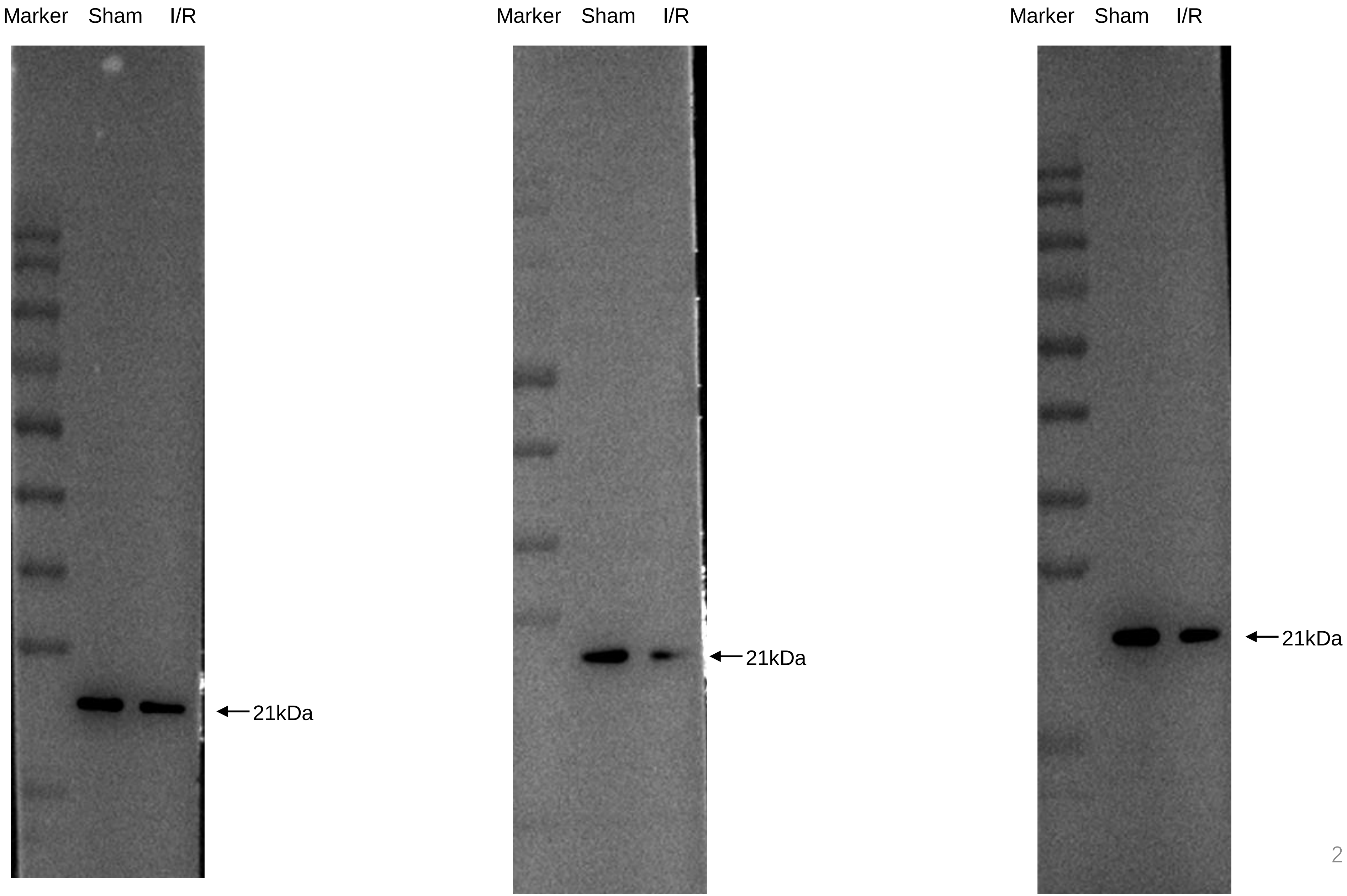

Figure1D ACSL4

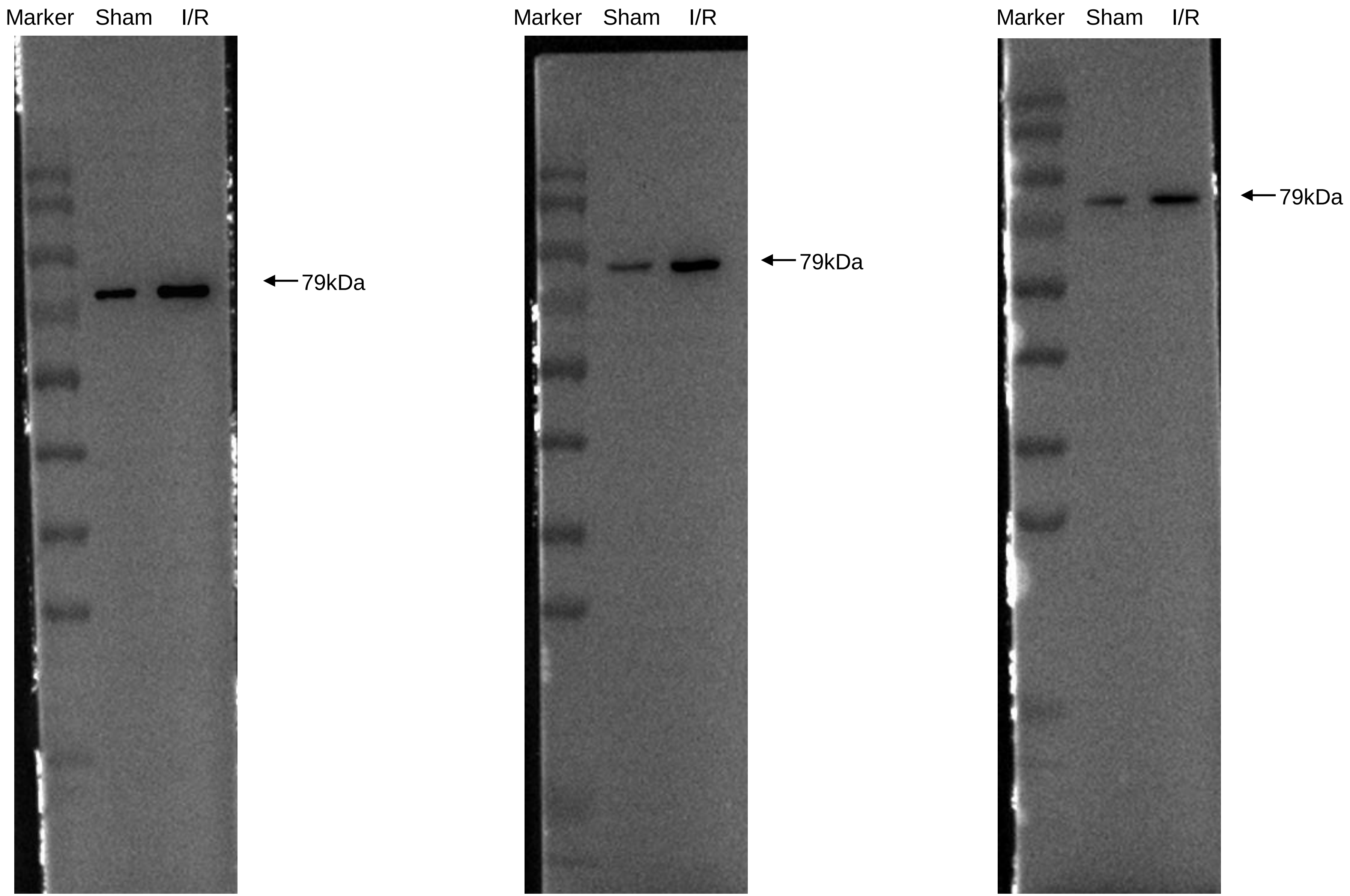

Figure1D NOX1

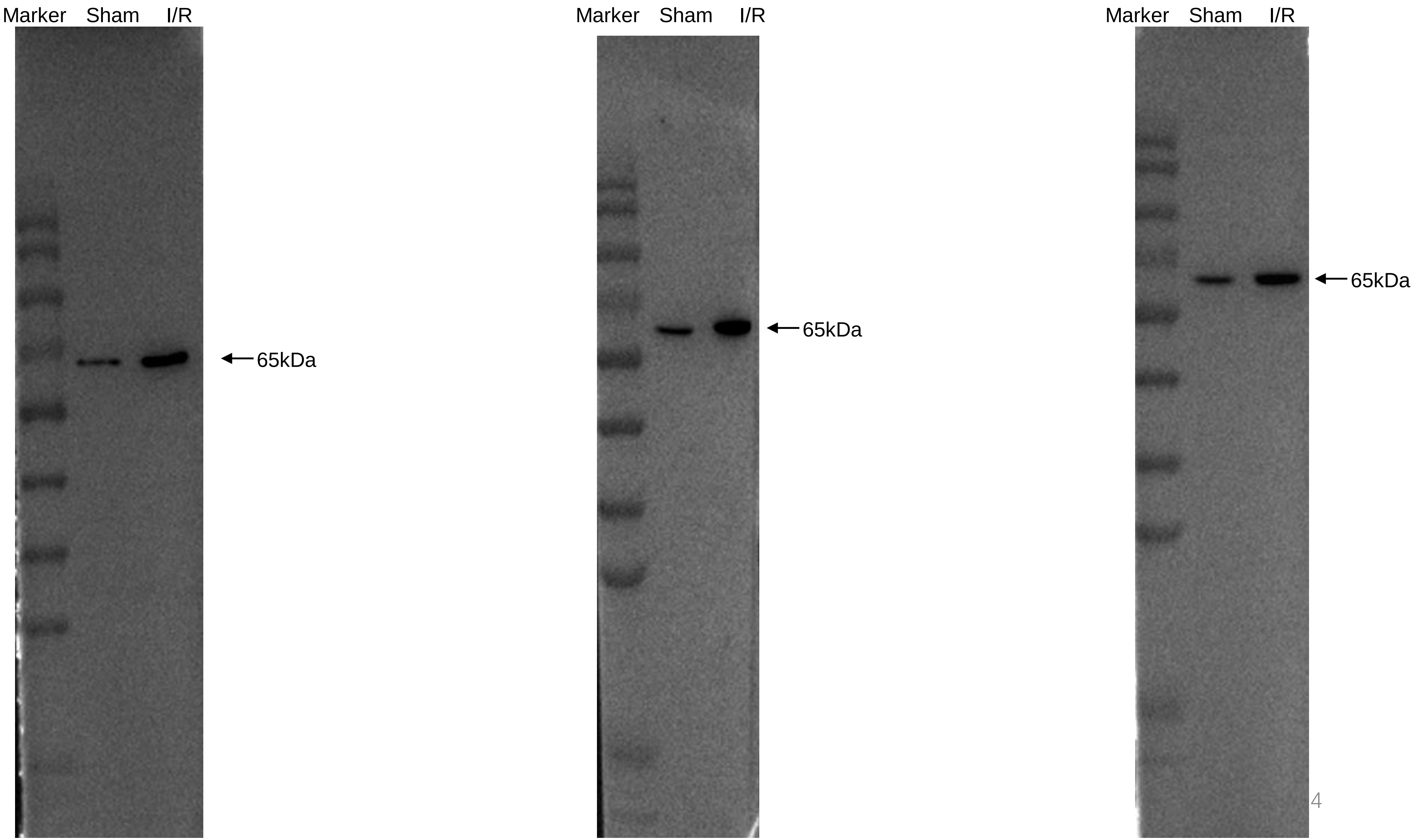

Figure1D COX2

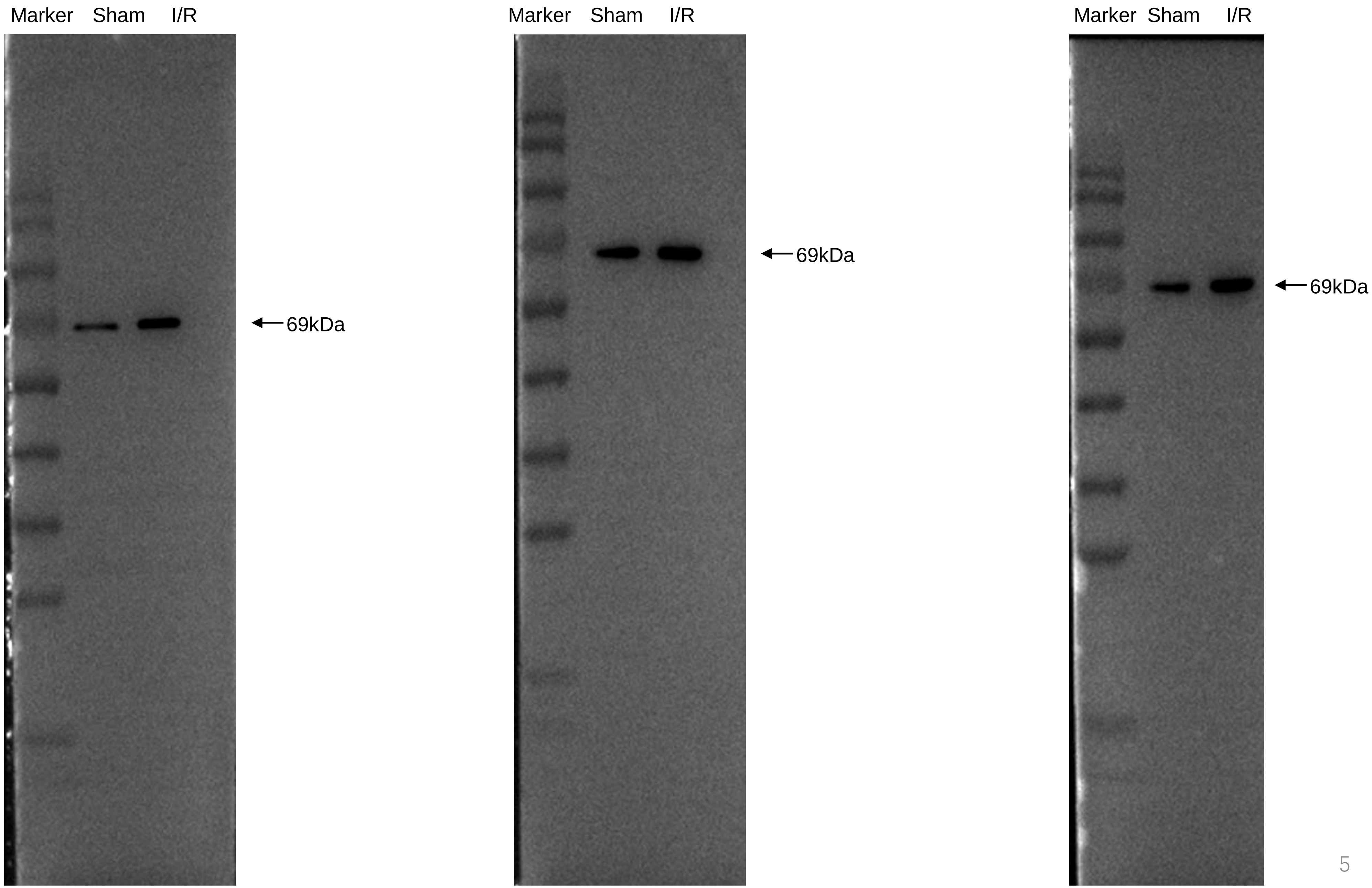

Figure1D GAPDH

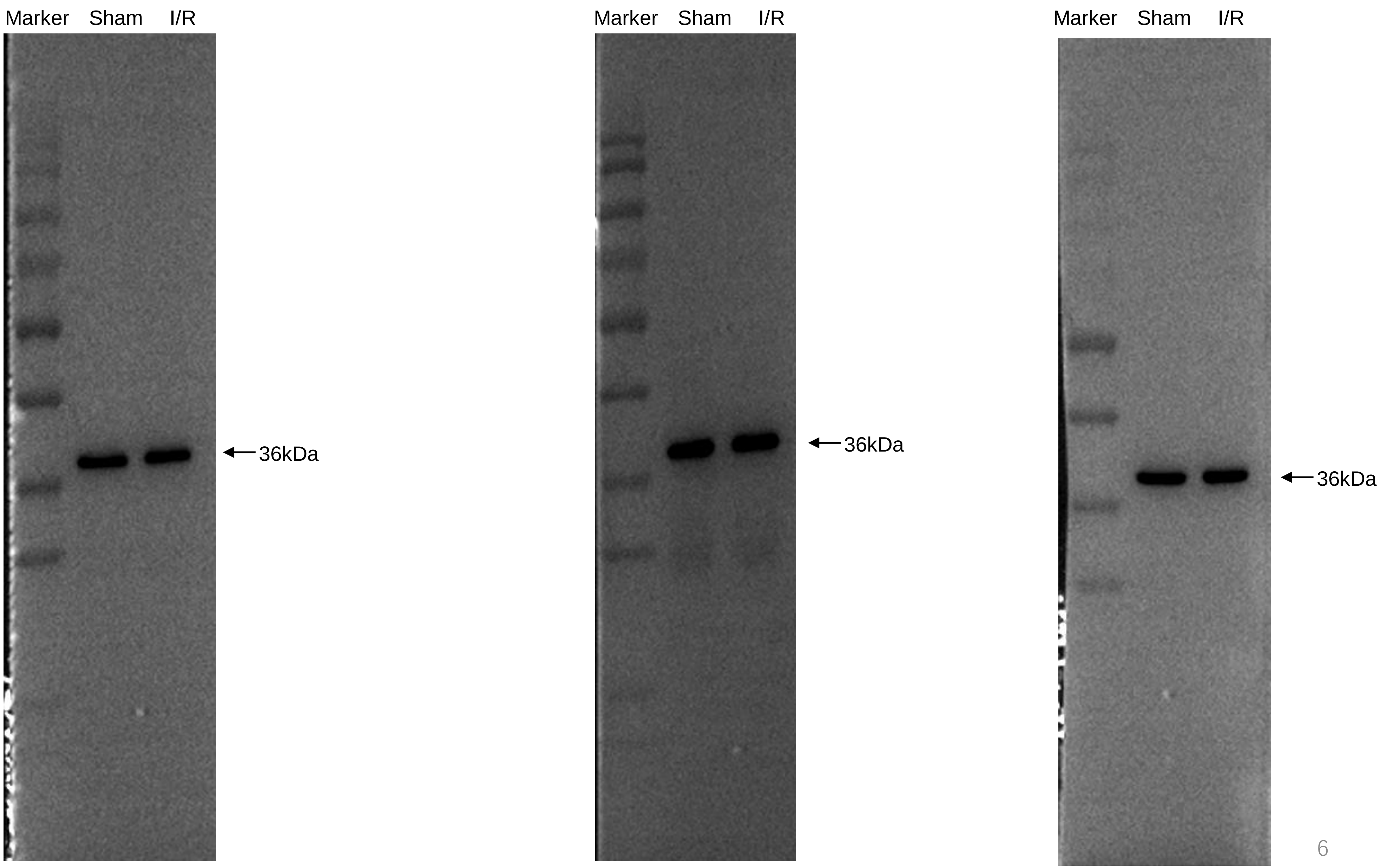

Figure3C GPX4

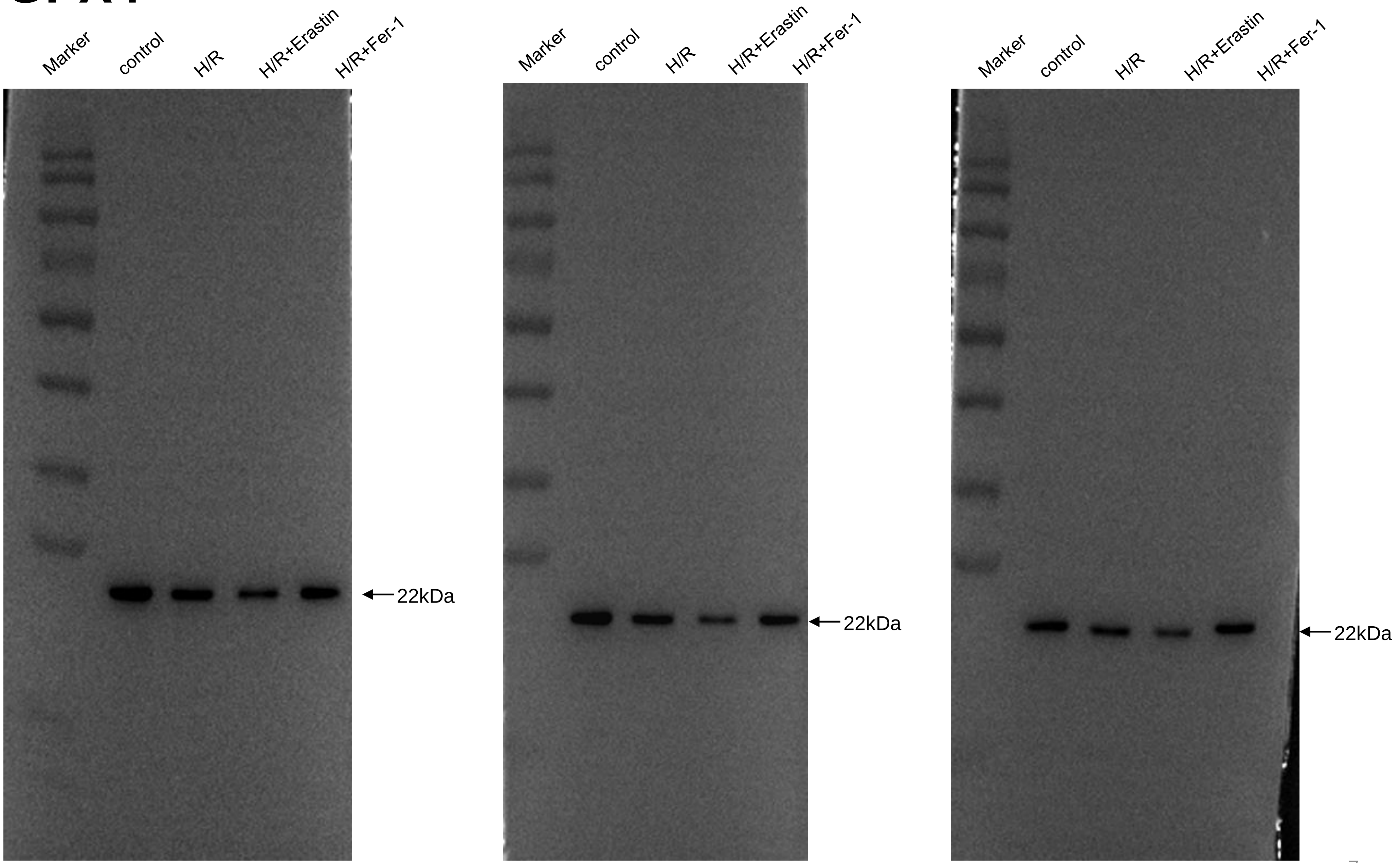

Figure3C FTH1

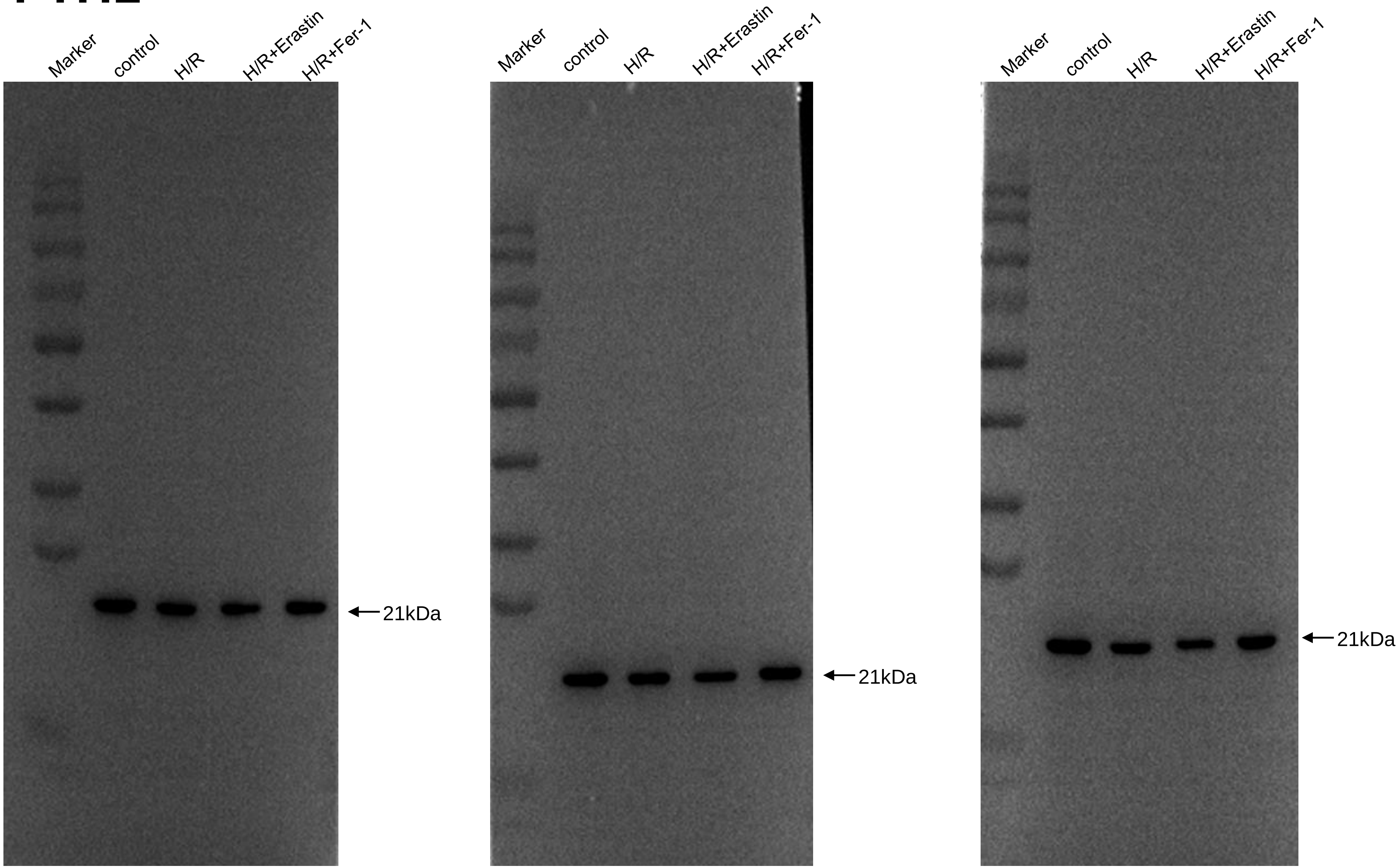

Figure3C ACSL4

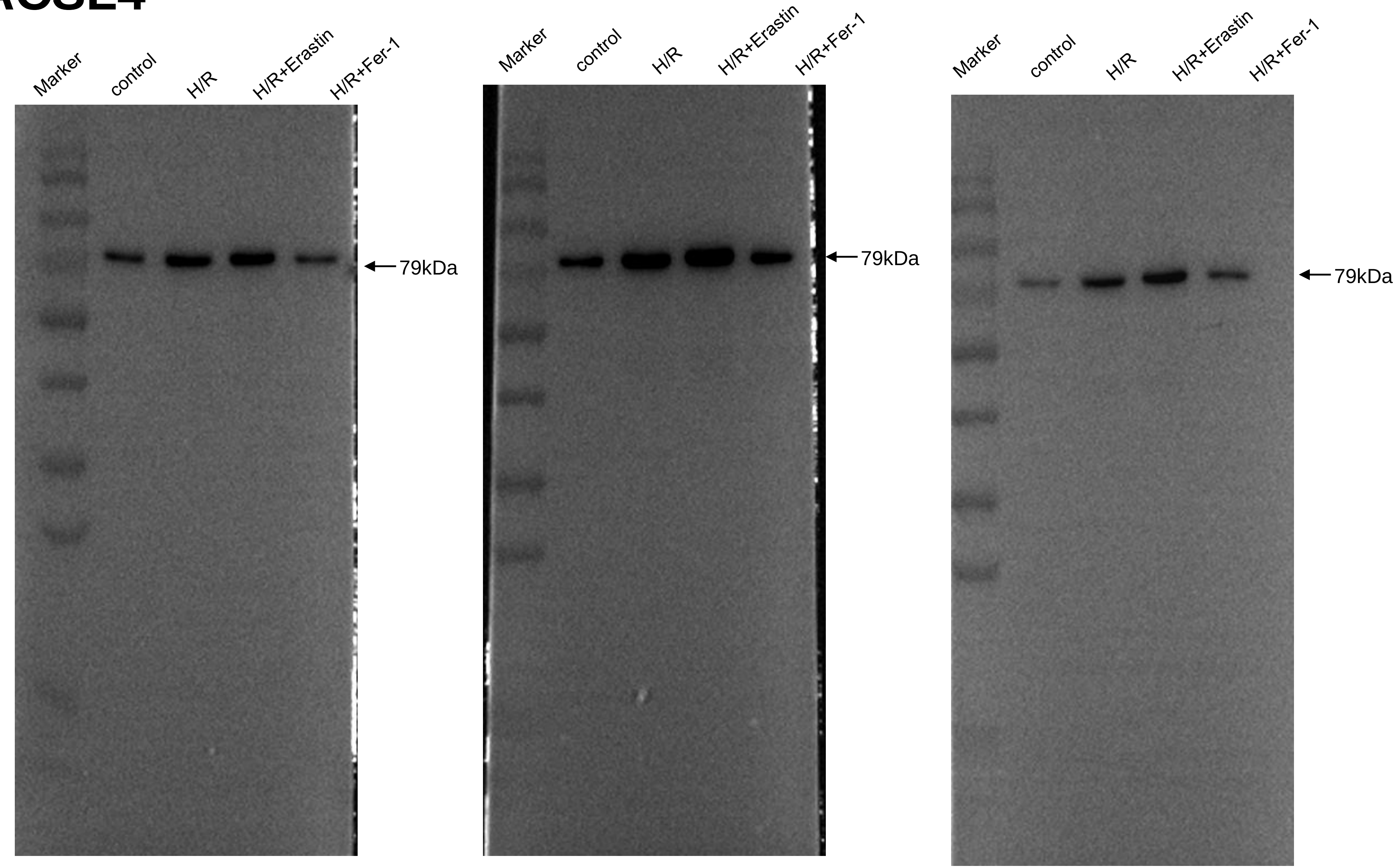

Figure3C NOX1

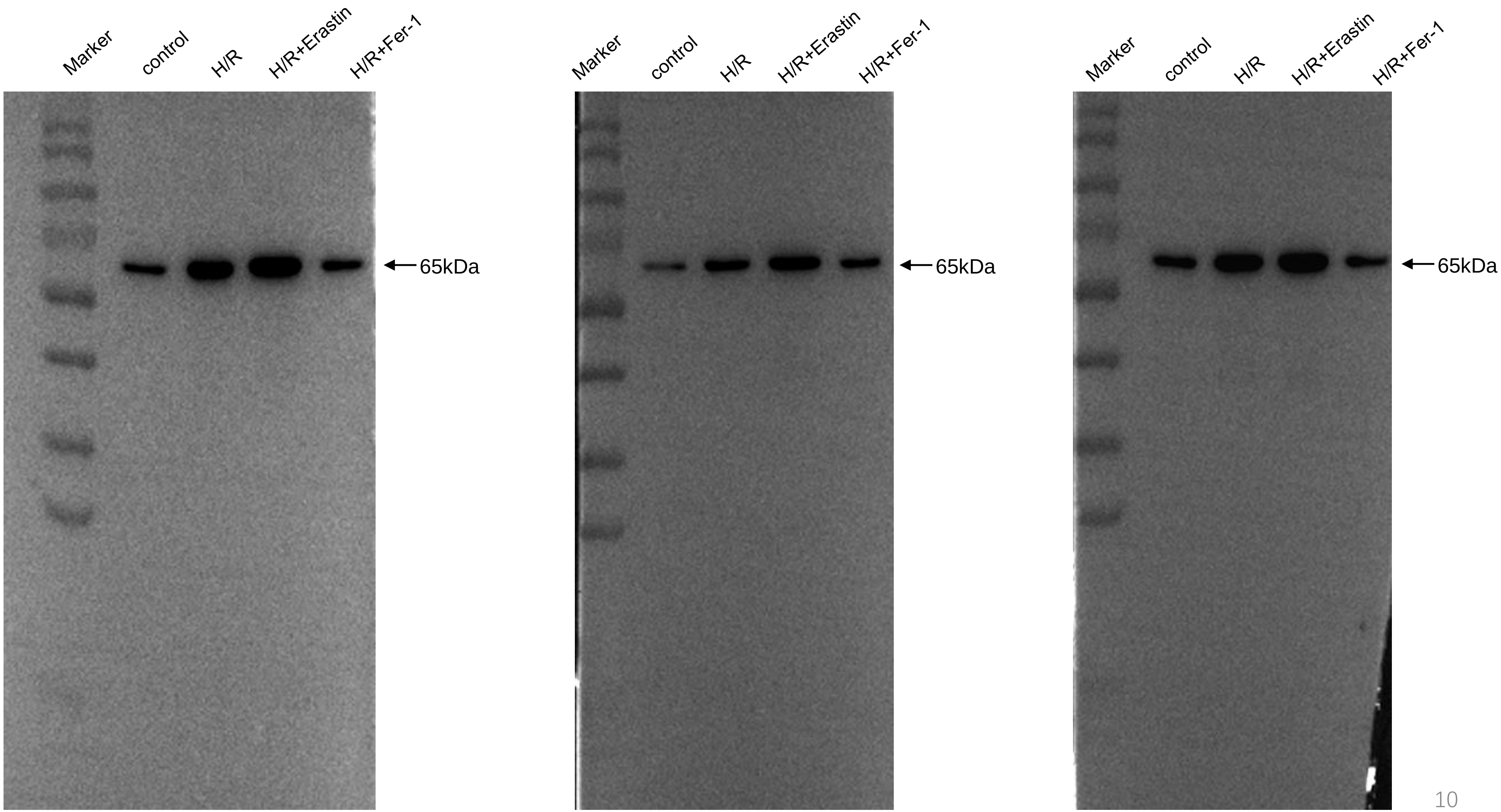

Figure3C COX2

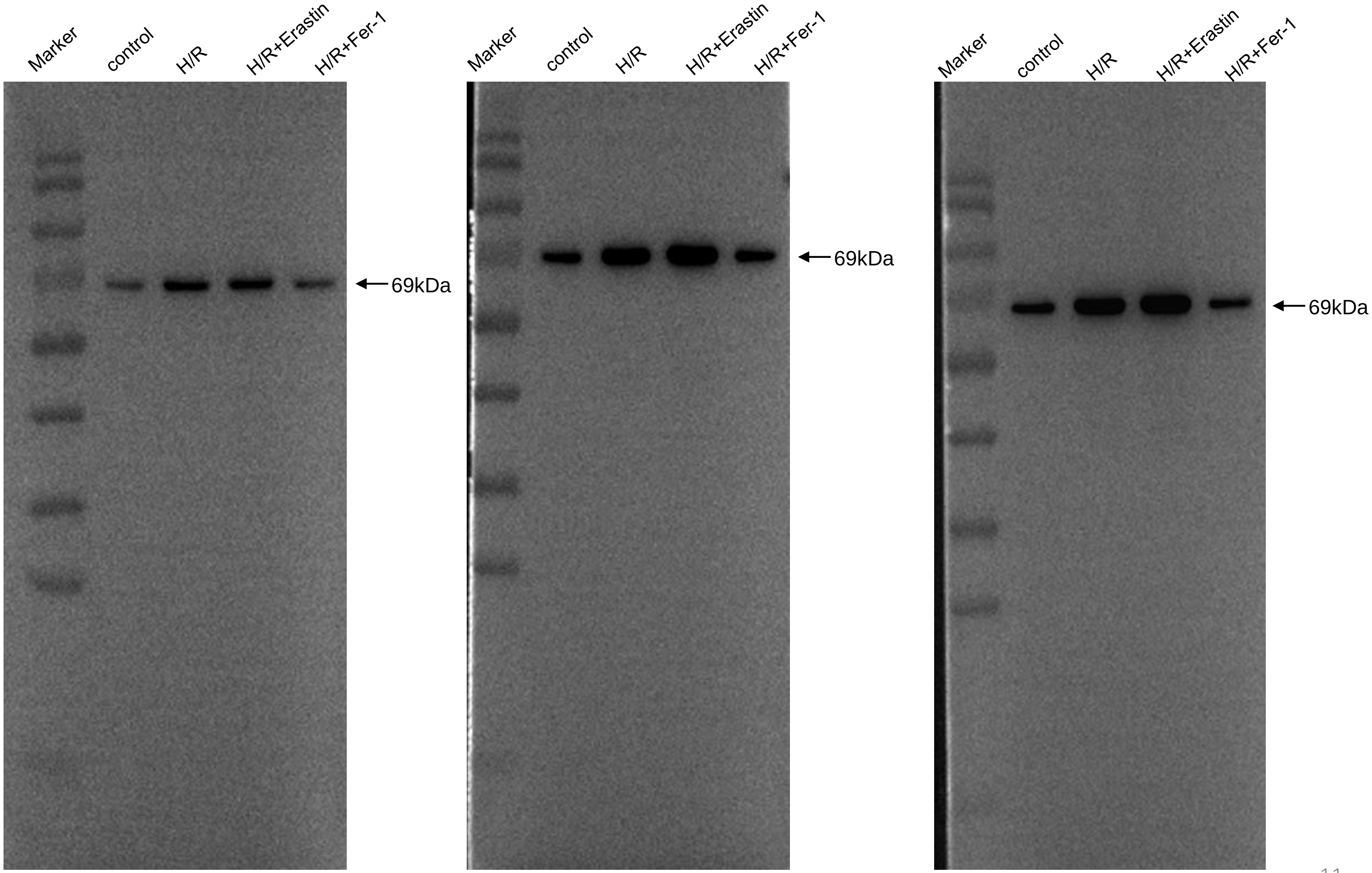

Figure3C GAPDH

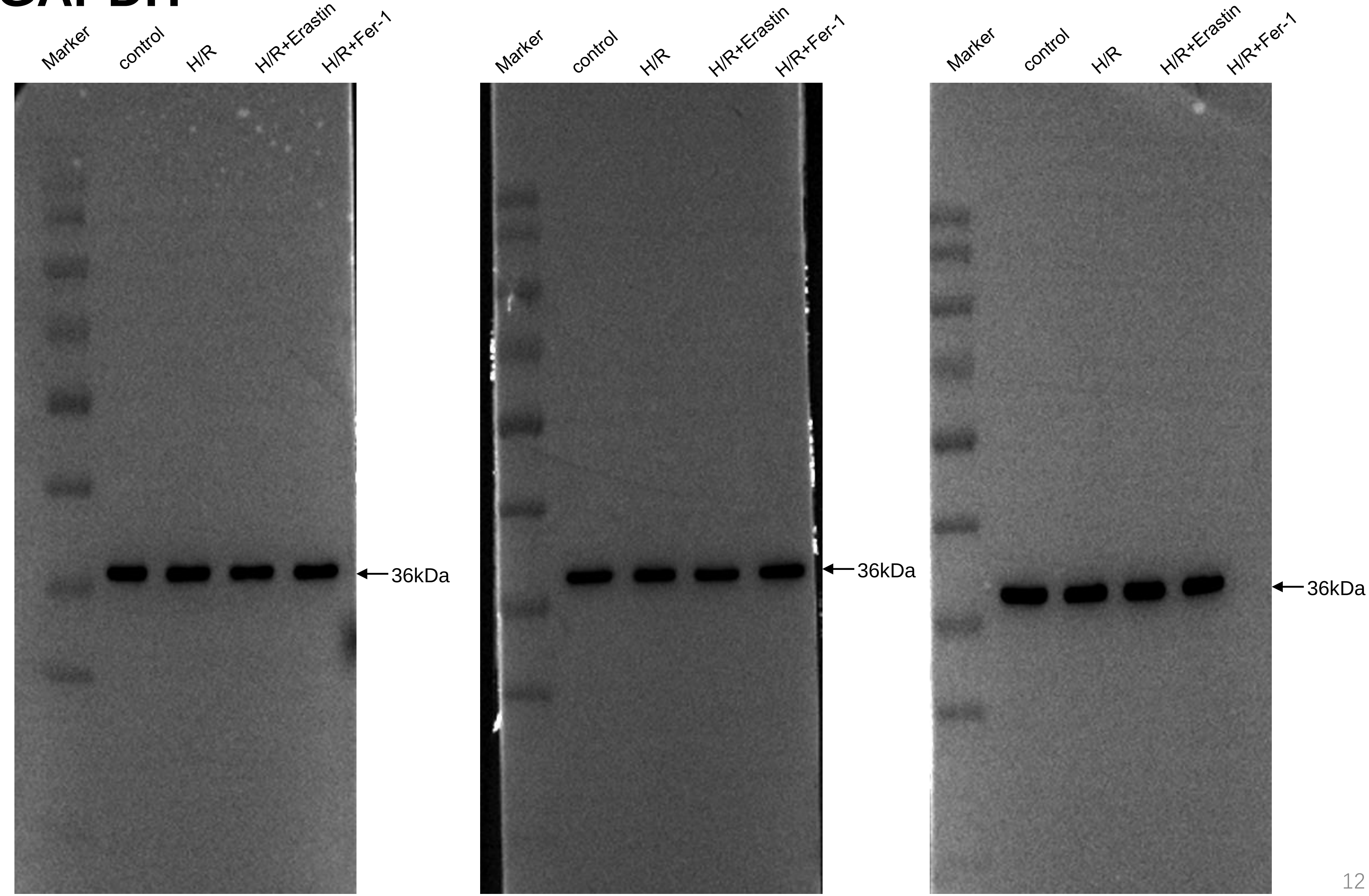

Figure4B GPX4

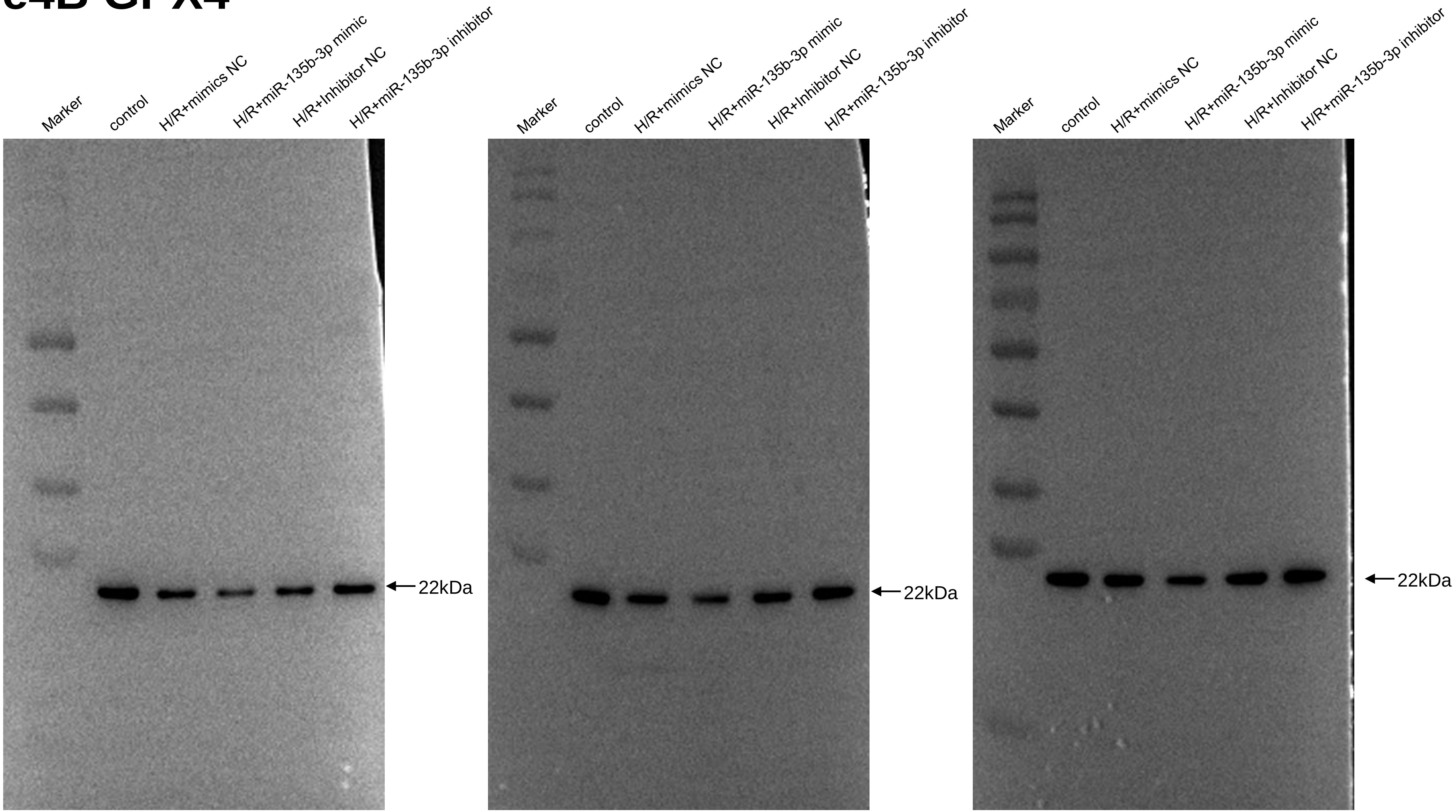

Figure4B GAPDH

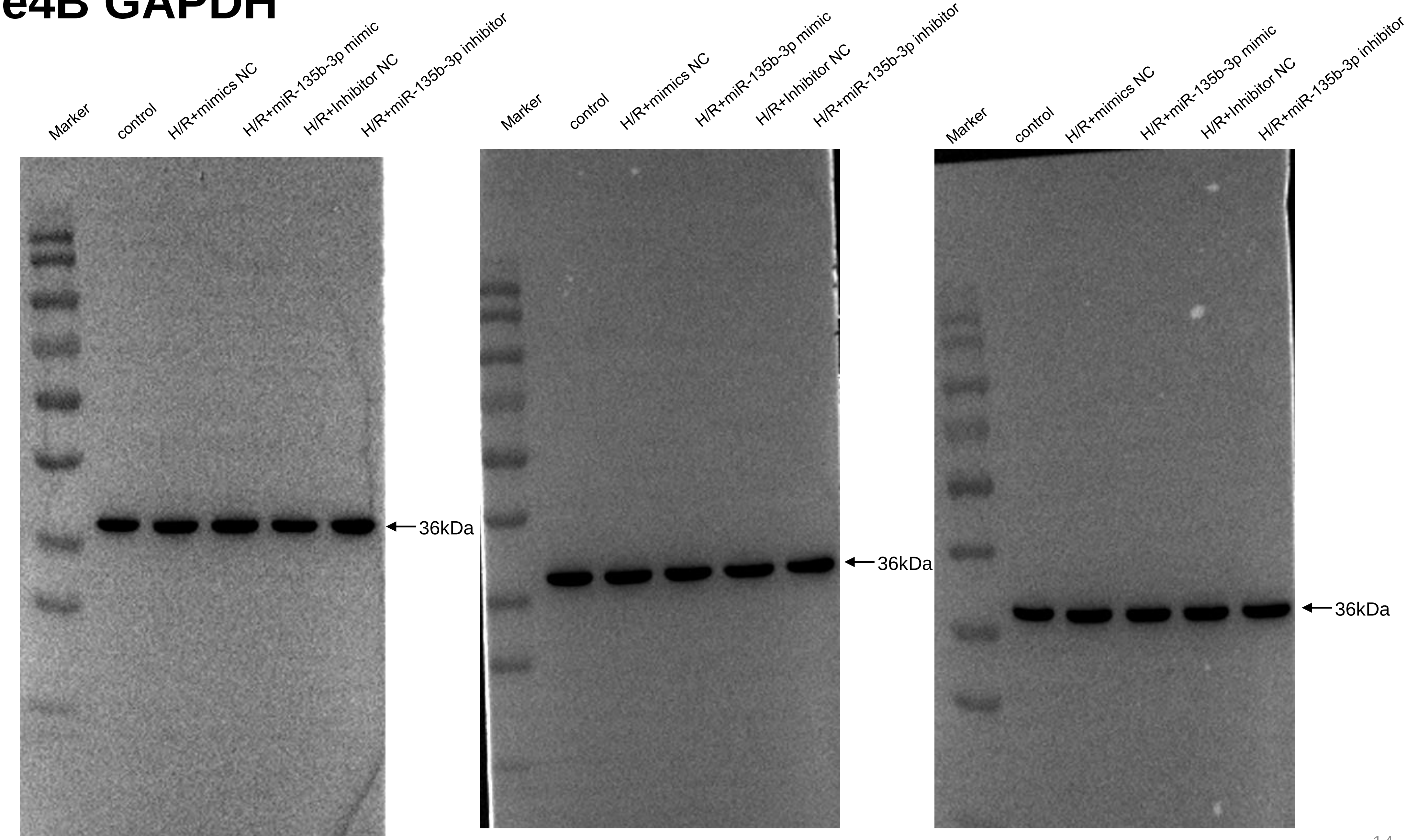

Figure5A GPX4

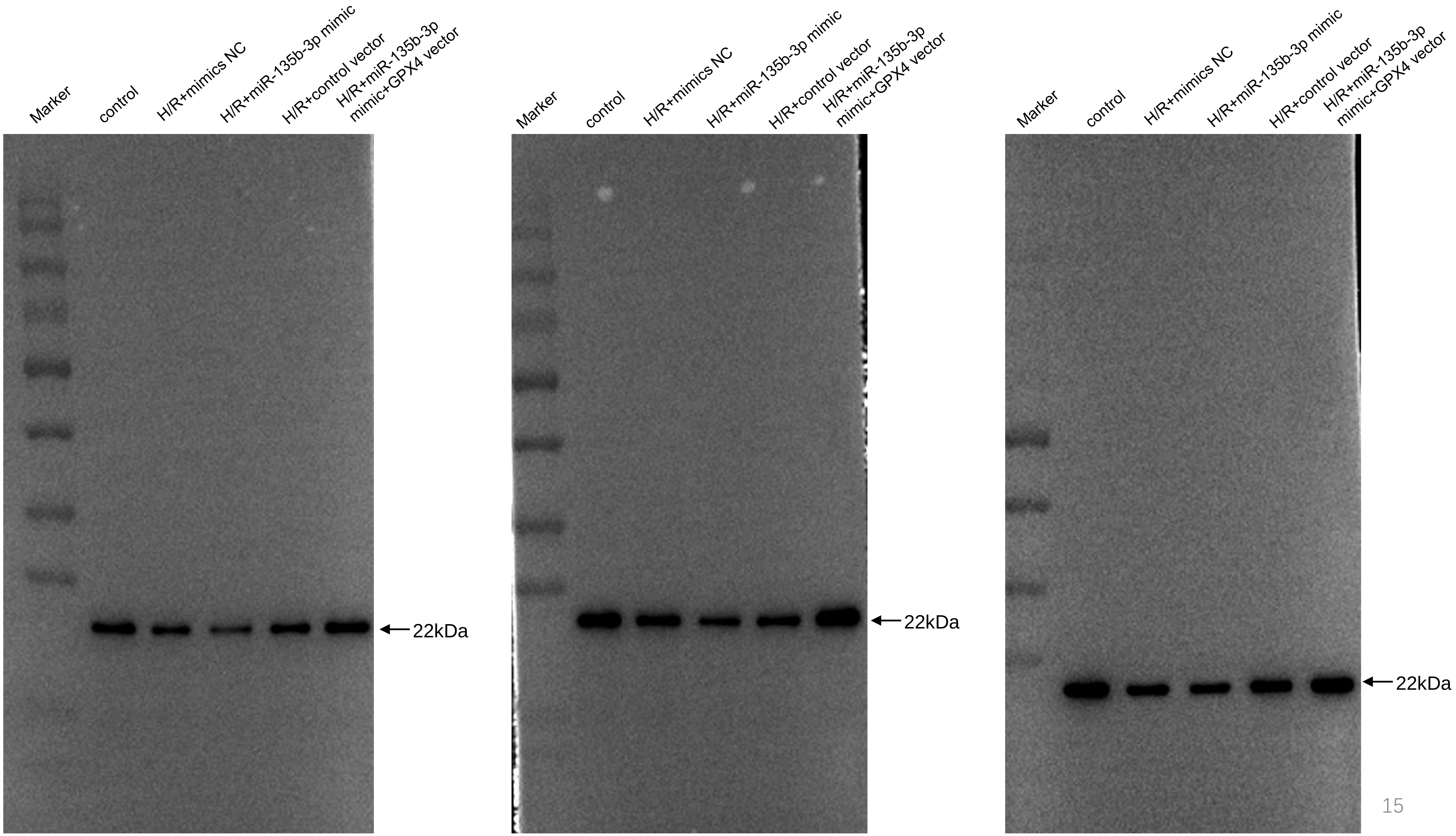

Figure5A FTH1

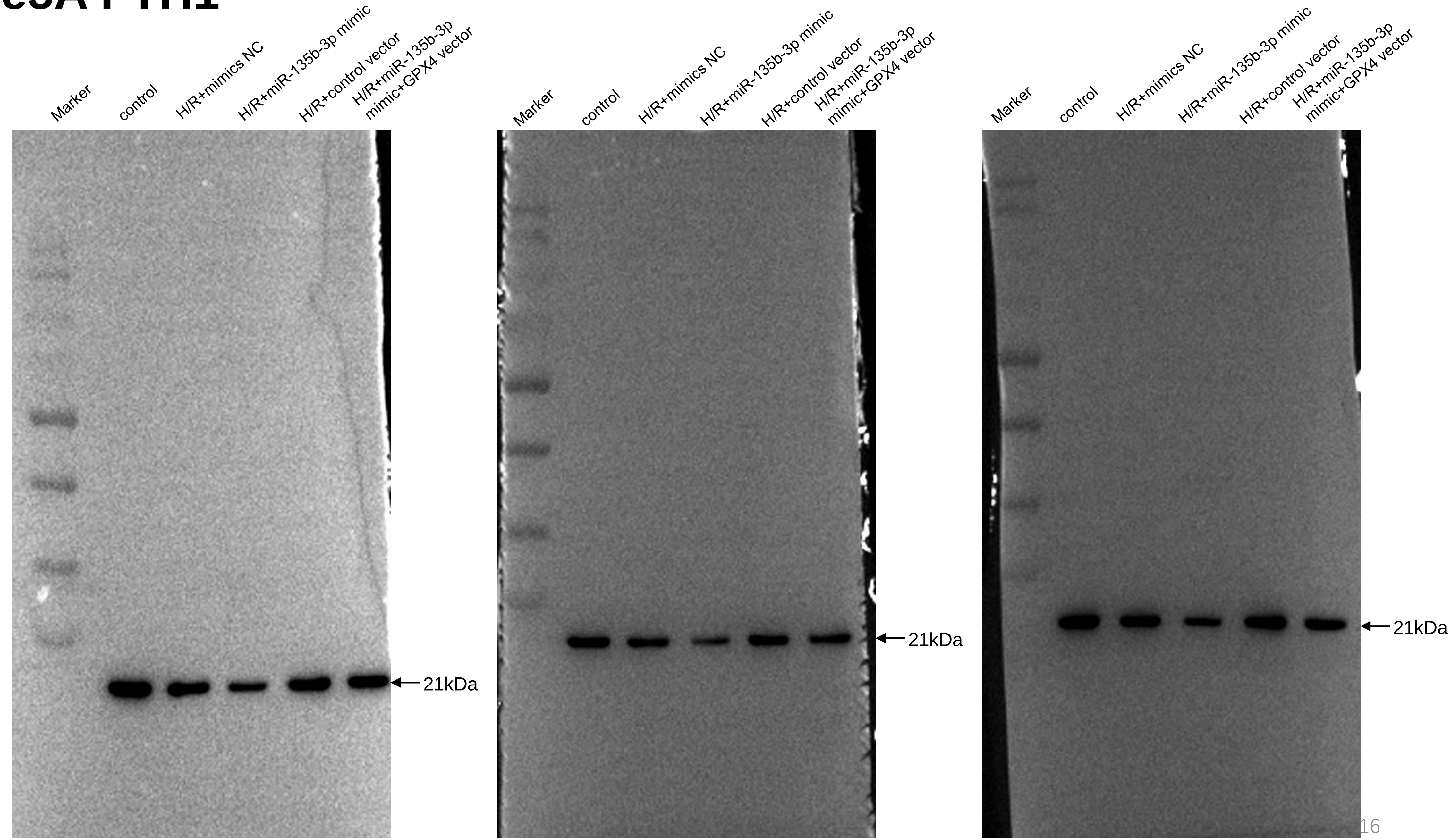

Figure5A ACSL4

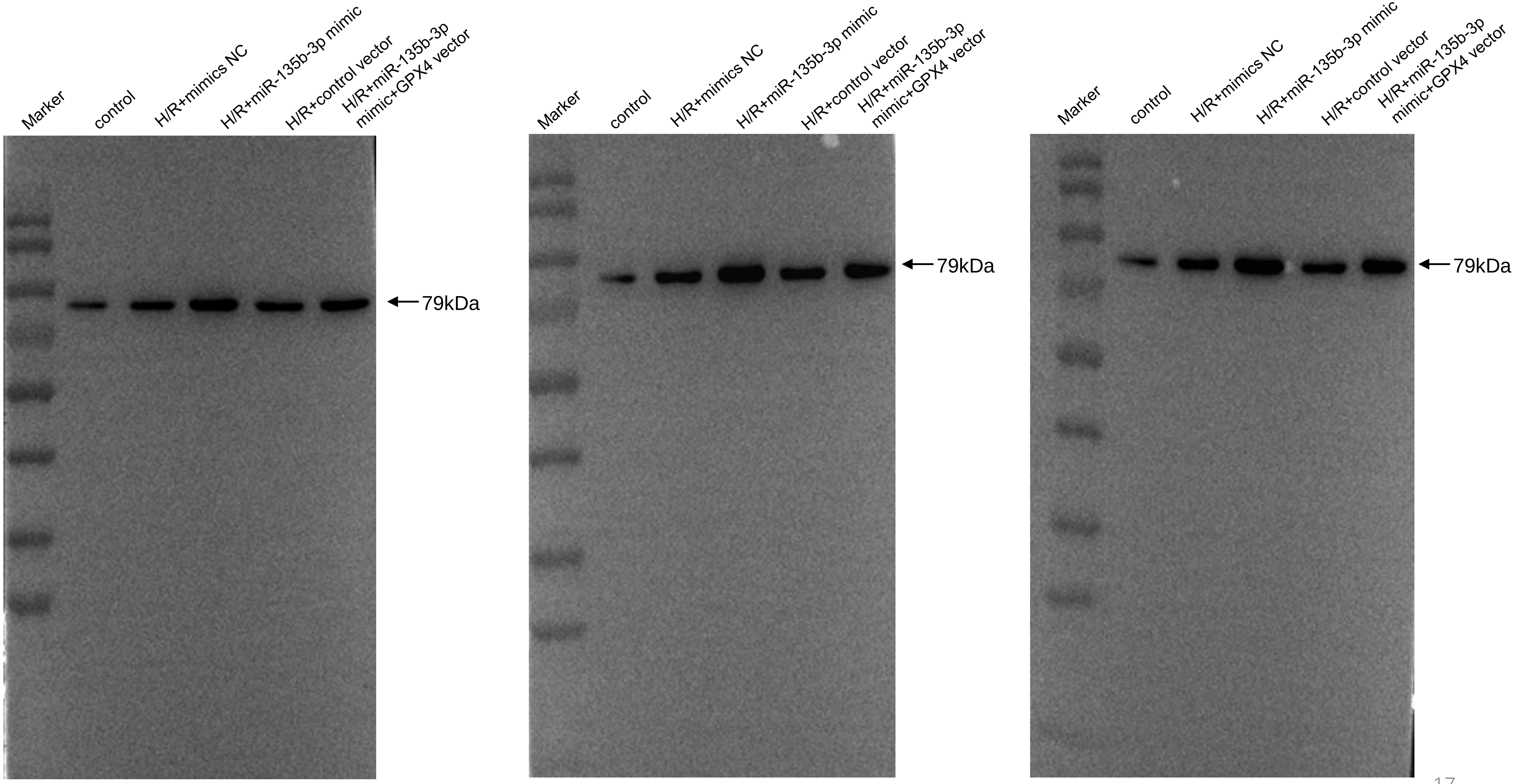

Figure5A NOX1

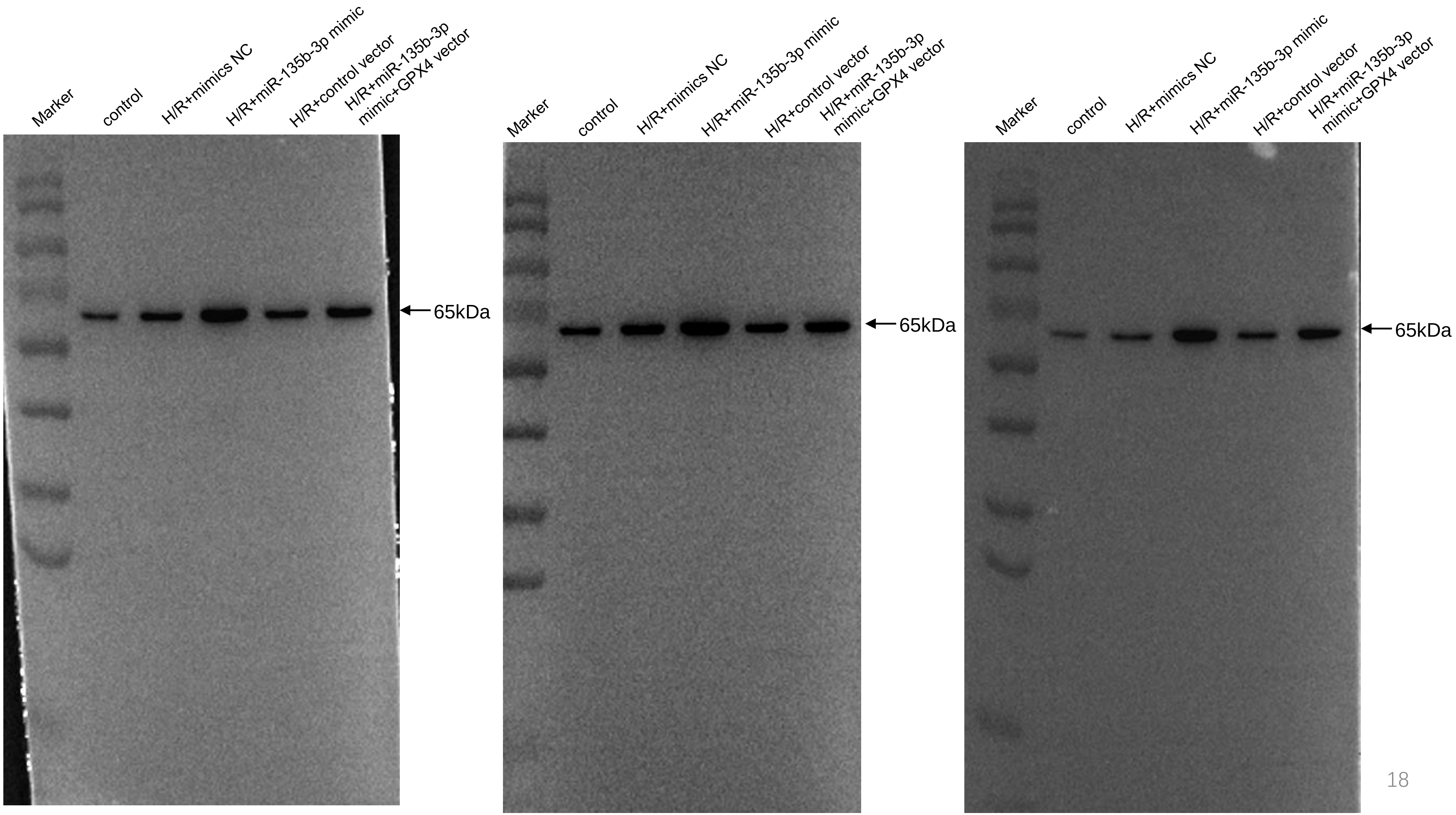

Figure5A COX2

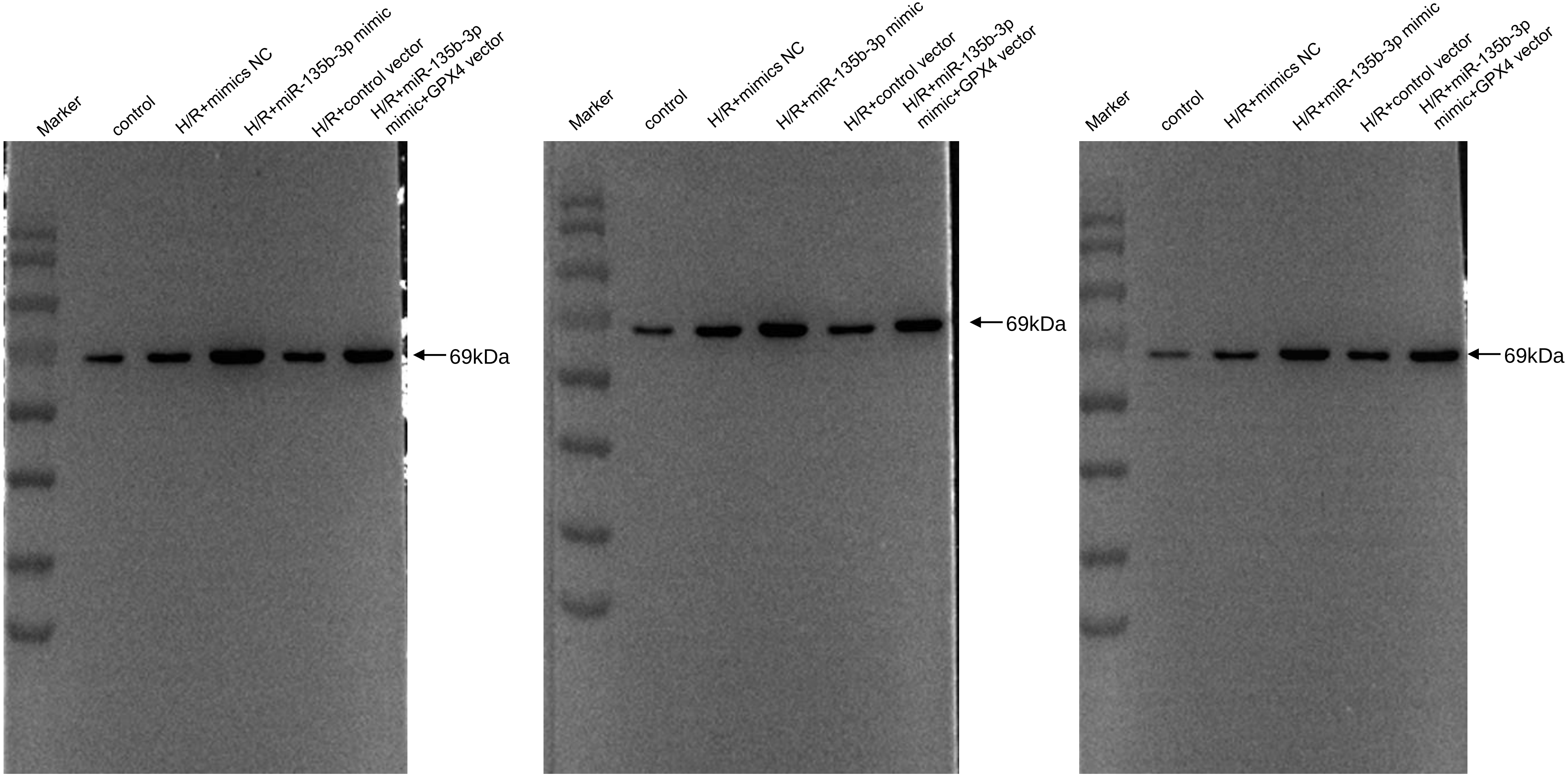

Figure5A GAPDH

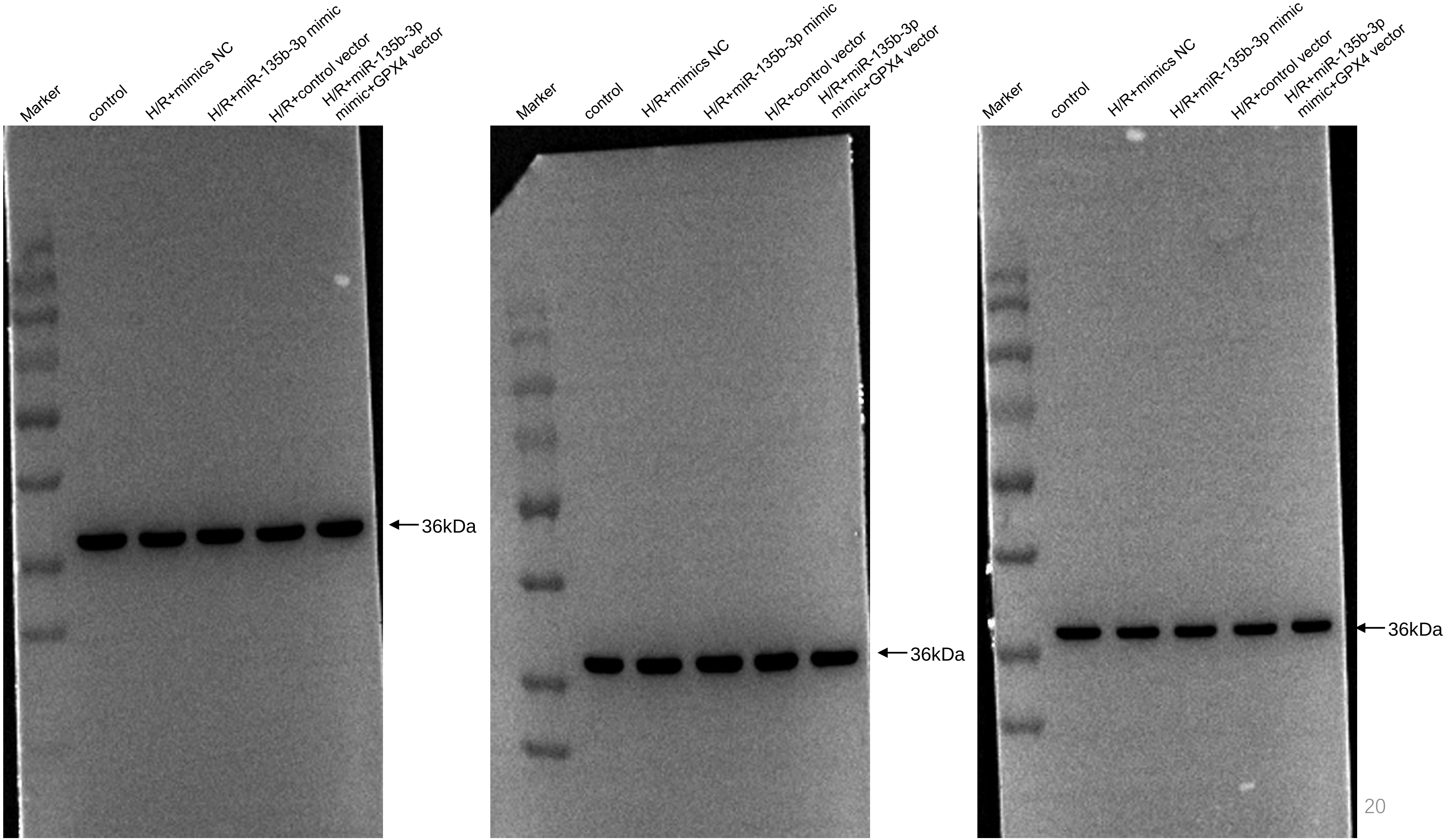

Figure6C GPX4

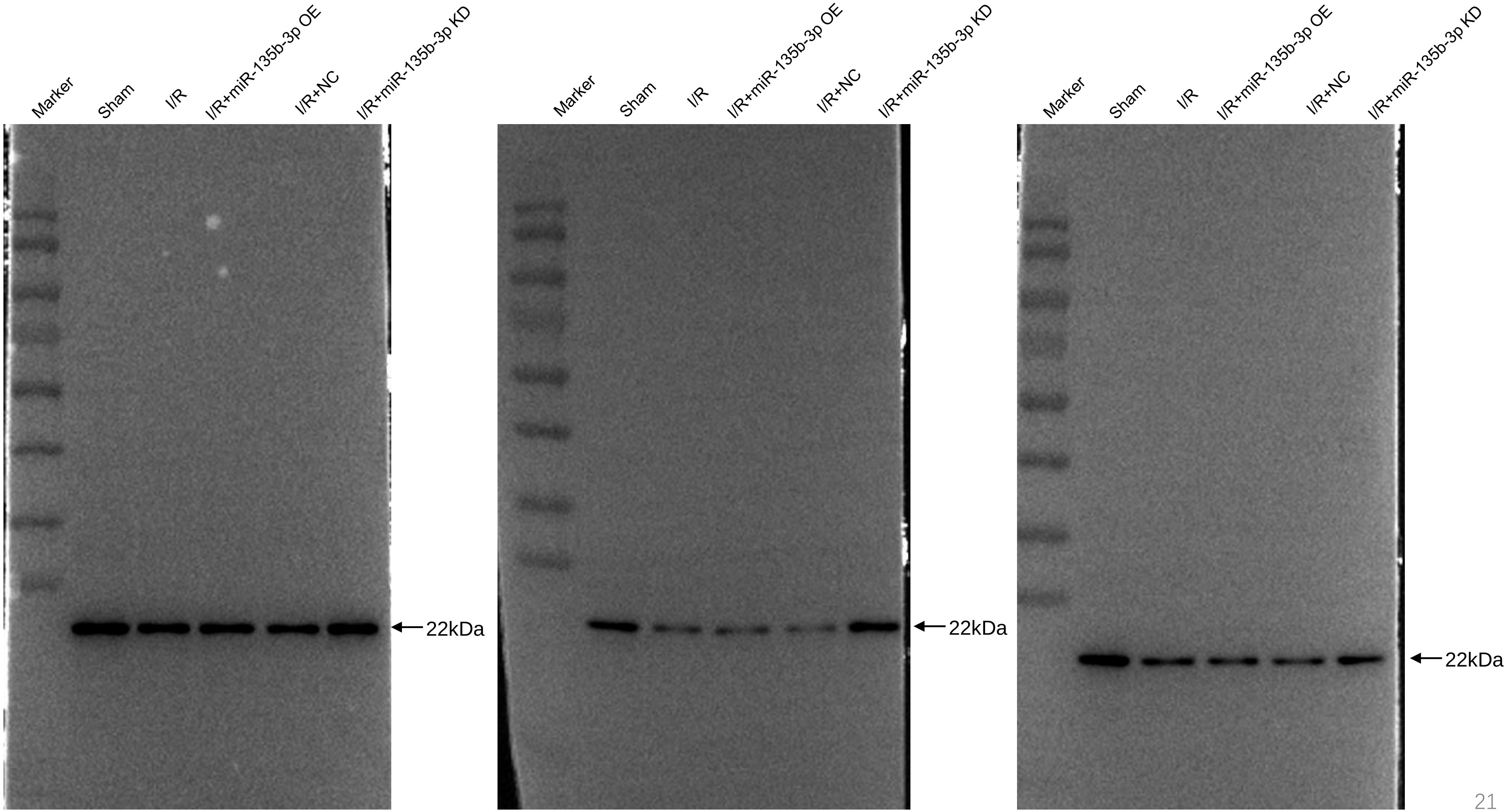

Figure6C GAPDH

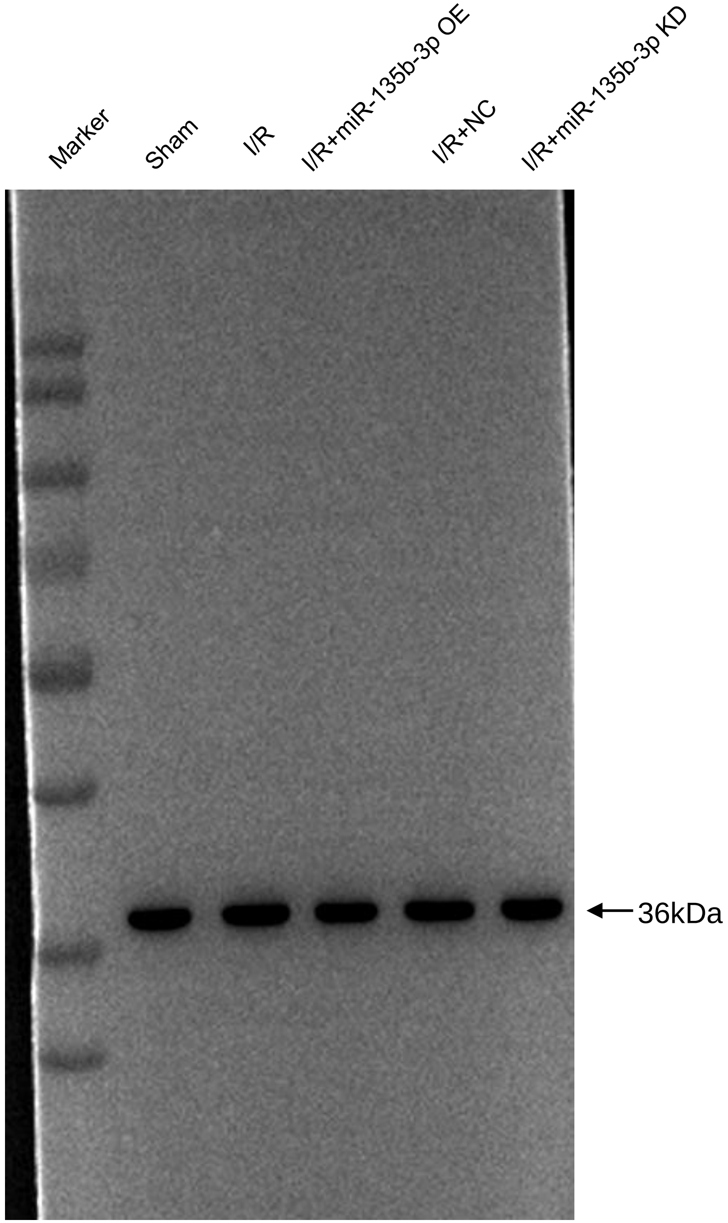

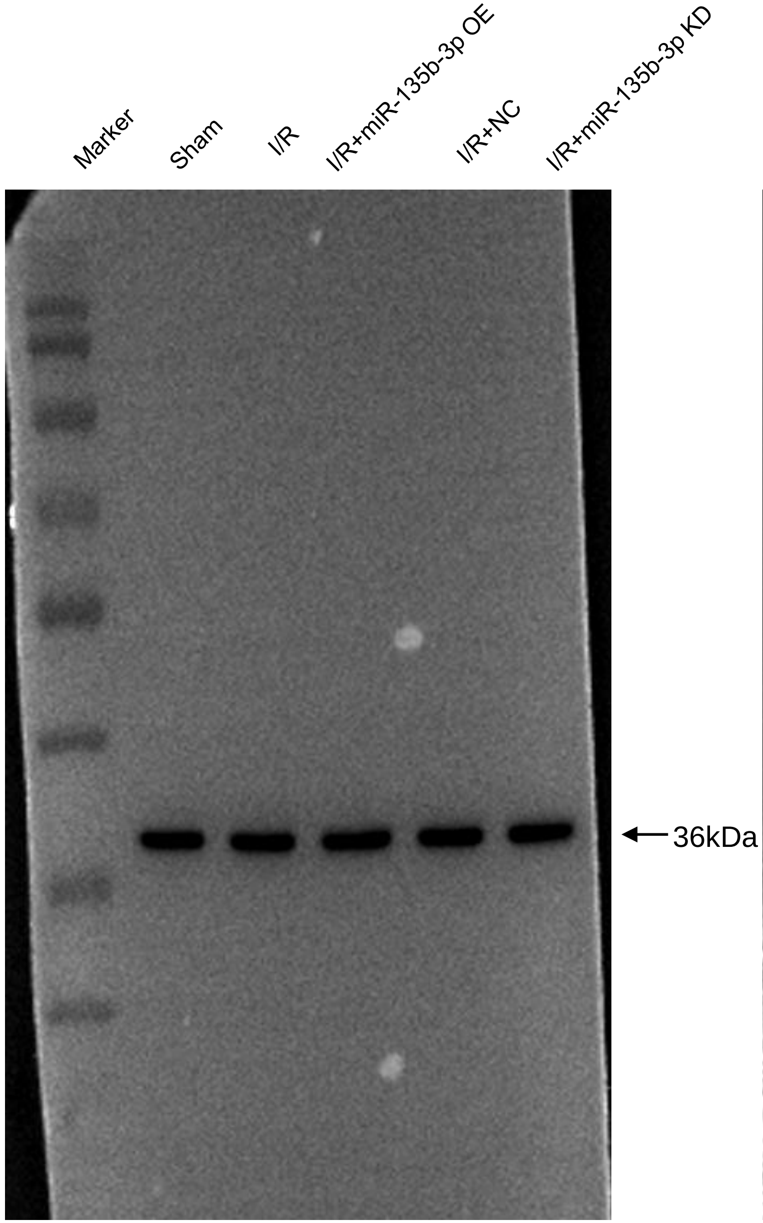

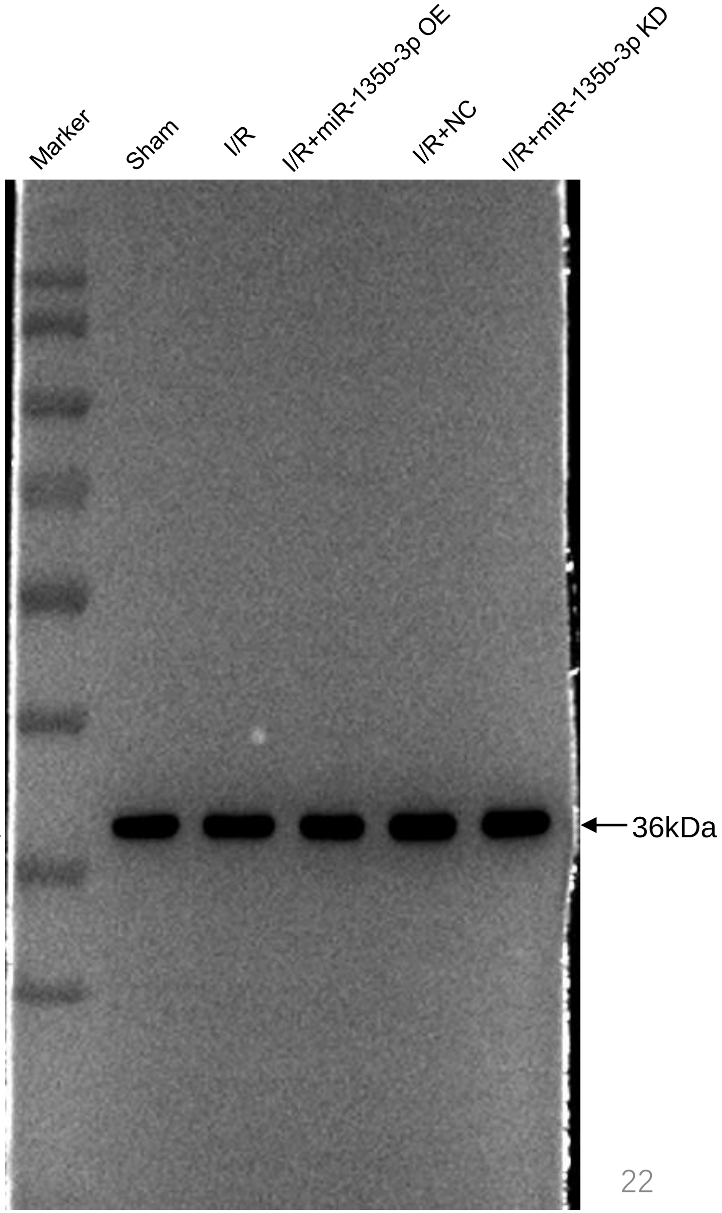

Supplement: Supplementary Figure 2 — The Full Scan WB images in the study. [file Image_2.pdf]
